# IA-DTPSO: A Multi-Strategy Integrated Particle Swarm Optimization for Predicting the Total Urban Water Resources in China

**DOI:** 10.3390/biomimetics10040233

**Published:** 2025-04-08

**Authors:** Zheyu Zhu, Jiawei Wang, Kanhua Yu

**Affiliations:** 1 School of Architecture, Chang’an University, Xi’an 710061, China; zhuzheyu1021@163.com; 2 College of Architecture, Xi’an University of Architecture and Technology, Xi’an 710055, China; 3 Xi’an International Science and Technology Cooperation Base: International Joint Research Center for Green Urban Rural and Land Space Smart Construction Technology Innovation, Xi’an 710000, China

**Keywords:** particle swarm optimization, Sobol sequence initialization, information acquisition strategy, Spearman’s correlation coefficient, tangent flight strategy, dimension learning strategy, three parameter grey combination optimization model, prediction of total urban water resources in China

## Abstract

In order to overcome the drawbacks of low search efficiency and susceptibility to local optimal traps in PSO, this study proposes a multi-strategy particle swarm optimization (PSO) with information acquisition, referred to as IA-DTPSO. Firstly, Sobol sequence initialization on particles to achieve a more uniform initial population distribution is performed. Secondly, an update scheme based on information acquisition is established, which adopts different information processing methods according to the evaluation status of particles at different stages to improve the accuracy of information shared between particles. Then, the Spearman’s correlation coefficient (SCC) is introduced to determine the dimensions that require reverse solution position updates, and the tangent flight strategy is used to improve the inherent single update method of PSO. Finally, a dimension learning strategy is introduced to strengthen individual particles’ activity, thereby ameliorating the entire particle population’s diversity. In order to conduct a comprehensive analysis of IA-DTPSO, its excellent exploration and exploitation (ENE) capability is firstly validated on CEC2022. Subsequently, the performance of IA-DTPSO and other algorithms on different dimensions of CEC2022 is validated, and the results show that IA-DTPSO wins 58.33% and 41.67% of the functions on 10 and 20 dimensions of CEC2022, respectively. Finally, IA-DTPSO is employed to optimize parameters of the time-dependent gray model (1,1,*r*,*ξ*,*Csz*) (TDGM (1,1,*r*,*ξ*,*Csz*)) and applied to simulate and predict total urban water resources (TUWRs) in China. By using four error evaluation indicators, this method is compared with other algorithms and existing models. The results show that the total MAPE (%) value obtained by simulation after IA-DTPSO optimization is 5.9439, which has the smallest error among all comparison methods and models, verifying the effectiveness of this method for predicting TUWRs in China.

## 1. Introduction

Optimization is the procedure of determining the most efficient solution for practical problems. For an optimization problem, it is necessary to first clarify the three basic elements of the problem: the number of decision variables, the optimization range of variables, and the objective function. At present, optimization techniques have been employed in various domains such as business, engineering, science, and medicine [1,2,3,4]. However, classical numerical methods struggle to offer accurate solutions for non-convex problems with non-linear constraints, leading to extended computation time [5]. Meta-heuristics are key tools for developing efficient optimizers that can effectively solve challenging real-world problems [6,7]. Metaheuristic algorithms (MAs) can traverse the solution space with effect in indeterminate environments to recognize global optima or find an approximate optimum [8]. This means that although MAs cannot guarantee an accurate solution, they can definitely generate an optimal solution [9]. MAs have high scalability and can be directly designed and implemented and can surmount challenges related to the enormous sophistication of mathematical inference [10,11]. Therefore, when traditional optimization techniques cannot handle exact solutions, MAs become an alternative method for quickly solving large-scale optimization problems [12]. MAs’ main features can be generalized as below:

- It is an approximate method that is not specific to a particular problem.
- It is a process of continuously learning towards the optimal solution through trial and error.
- Demonstrates significant multi-functionality and robustness.
- It is an optimization logic used to determine approximate solutions to complex global optimization problems.

All population-based MAs possess these characteristics, with differences only in the use of operators and mechanisms. In addition, MAs also include two essential search tactics, namely ENE [13,14]. Exploration is the ability to search the solution space on a global scale, which is associated with averting local optimality and solving traps in local optimality. Exploitation is about making optimal decisions from promising solutions in the vicinity to increase MAs’ local quality [15]. Therefore, the key to whether a MA has excellent performance depends on whether an appropriate balance can be achieved between these two strategies. This typically involves how to use search operators to effectively extract and utilize information, thereby generating more promising solutions to problems [16].

Thirty years ago, MAs represented by PSO [17] gained widespread recognition in the research community, and more and more MAs rapidly emerged under its influence. So far, thousands of MAs have been made public. According to different sources of inspiration, MAs can be broadly divided into four categories: swarm-behavior inspired, human-behavior inspired, evolution-phenomena inspired, and nature-science-phenomena inspired.

(i) Swarm-behavior inspired: Swarm-behavior-inspired algorithms are techniques that mimic collaborative behavior in biological social systems to solve problems. They organize a large number of simple individual units (such as ants, bees, bird swarm agents) together, allowing them to interact and learn in complex environments, and jointly search for optimal solutions. In recent years, newly proposed population-based algorithms include: Whale Optimization Algorithm (WOA) [18], Northern Goshawk Optimization (NGO) [19], Bottlenose Dolphin Optimizer (BDO) [20], Nutcracker Optimization Algorithm (NOA) [21], Mantis Search Algorithm (MSA) [22], Genghis Khan Shark Optimizer (GKSO) [23], Black-winged kite algorithm (BKA) [24], Secretary Bird Optimization Algorithm (SBOA) [25], and Horned Lizard Optimization Algorithm (HLOA) [26].
(ii) Human-behavior inspired: Human-behavior-inspired algorithms typically draw inspiration from human creativity, artistic thinking, and problem-solving approaches, simulating the process of humans making a series of decisions through team collaboration. In recent years, this type of algorithm includes: Enterprise Development Optimizer (EDO) [27], Hiking Optimization Algorithm (HOA) [28], Great Wall Construction Algorithm (GWCA) [29], Football Team Training Algorithm (FTTA) [30], Alpine Skiing Optimization (ASO) [31], Information Acquisition Optimizer (IAO) [32], Adolescent Identity Search Algorithm (AISA) [33], and Information Decision Search Algorithm (IDSE) [34].
(iii) Evolution-phenomena inspired: Evolution-phenomena-inspired algorithms are mainly a type of computational technology that draw inspiration from biological evolution theory. These mainly include Genetic Algorithm (GA) [35], Genetic Programming (GP) [36], Evolutionary Programming (EP) [37], Evolutionary Strategy (ES) [38], Differential Evolution (DE) algorithm [39], Biogeography-based optimization (BBO) [40], Clonal Selection Algorithm (CSA) [41], and Alpha Evolution (AE) [42].
(iv) Nature-science-phenomena inspired: Nature-science-phenomena-inspired algorithms based on natural science phenomena mainly come from observations of natural phenomena and scientific laws in various fields. The latest achievements in this research direction mainly include: Tangent Search Algorithm (TSA) [43], Kepler Optimization Algorithm (KOA) [44], Exponential- Trigonometric Optimization (ETO) algorithm [45], Artemisinin Optimization (AO) algorithm [46], Weighted Average Algorithm (WAA) [5], Newton-Raphson-based Optimizer (NRBO) [47], Polar Lights Optimization (PLO) [48], and FATA morgana algorithm (FATA) [49].

In addition to these classic MAs, many improved versions of MAs have emerged in this field and been applied in various practical applications. Yan et al. [50] developed an enhanced human memory optimizer to solve engineering optimization problems. Hu et al. [51] studied a multi-strategy DE algorithm for the smooth path planning of multi-scale robots and obtained a motion path with higher smoothness. Gobashy et al. [7] used WOA to solve the problem of spontaneous potential energy anomalies caused by 2D tilted plates of infinite horizontal length. Li et al. [52] proposed an improved seagull optimizer for fault location in distribution networks. Jamal et al. [53] proposed an improved Pelican optimization algorithm to solve non-convex stochastic optimal power flow problems in power systems, thereby reducing generation costs and emissions.

Although the successive emergence of various new MAs has added great vitality to the field of intelligent optimization, existing MAs still have several limitations, which can be generalized as below:

- Difficulty in achieving the optimal balance of ENE, resulting in MAs to local optimum.
- Multiple operators are typically used to approximate the optimum, complicating the search scenario.
- Performance degradation in high-dimensional search space.

As one of the classic MAs, PSO has hundreds or thousands of improved versions, but it is difficult to select the best from these improved versions due to the No Free Lunch (NFL) theorem that all MAs have to rely on [54]. This theorem emphasizes that no MA can be universally applicable to all types of problems. That is to say, different MAs may perform better for specific types of problems but may not be as effective for other types of problems. Furthermore, search operators’ basis vectors in PSO typically determine the starting point of the search and are sampled straightforwardly from the solution set instead of being adaptively selected. In addition, particles overly rely on obtaining information from two historical best positions while lacking the capacity to gain more information from other particles. Finally, PSO still exposes the imbalance of ENE. In response to the shortcomings of PSO mentioned above and combined with the NFL theorem, this study proposes a multi-strategy improved PSO (IA-DTPSO), which is based on the information acquisition strategy and involves four other improvement strategies for targeted auxiliary improvement. Compared with a large number of existing PSO variants, this method has a more novel structure and refined update method.

In recent years, combining MAs with predictive models has become a hot topic. However, existing prediction models have the characteristics of slow technological updates and slow application of new technologies. Usually, these models are mostly based on historical data, which makes it difficult to cope with complex changes in the future, thereby affecting prediction accuracy and efficiency. At present, this field has achieved relatively satisfactory optimization results by utilizing various hybrid MAs or other machine learning methods to process models. However, these methods often have low universality, and there is still room for improvement in terms of prediction accuracy and efficiency. In order to overcome these limitations, this study combines the proposed IA-DTPSO with the simulation and prediction of China’s TUWRs. Through the distinctive update method of PSO variants, the parameters of TDGM (1,1,*r*,*ξ*,*Csz*) are optimized step by step to achieve the solution of the simulation and prediction problem of China’s TUWRs.

This study’s main contributions are as below:

(i) A multi-strategy PSO with information acquisition, referred to as IA-DTPSO, is proposed and the entire optimization process is modeled.
(ii) The good ENE ability of IA-DTPSO is validated on CEC2022.
(iii) IA-DTPSO is compared with 11 other algorithms on different dimensions of CEC2022, verifying the superiority of IA-DTPSO.
(iv) IA-DTPSO and seven other algorithms are employed to optimize parameters of TDGM (1,1,*r*,*ξ*,*Csz*) and applied to predict TUWRs in China. In addition, the IA-DTPSO optimized model is compared with three existing models, and the results indicate that the model optimized by IA-DTPSO achieves the minimum error among the four error evaluation metrics in both comparisons.

This study’s remaining parts are arranged as below: Section 2 reviews PSO’s basic framework. Section 3 gives an optimization model for IA-DTPSO. Section 4 analyzes and discusses the experimental results of IA-DTPSO on CEC2022. Section 5 utilizes the proposed IA-DTPSO to optimize TDGM (1,1,*r*,*ξ*,*Csz*) and applies it to simulate and predict TUWRs in China. Section 6 provides a summary of this study and prospects for the future.

## 2. The Classic PSO

PSO [16] regards bird flocks as a group of particles with self-activity trajectories, and the activity trajectories of particles depend on their velocity *v* and position *x*. Then, the calculation formula for *v* at time *t* + 1 is shown in Equation (1) [16]:

(1) $$v_{i}^{t+1}=\omega \times v_{i}^{t}+c_{1}\times r_{1}\times ({\mathrm{PB}}_{i}^{t}-x_{i}^{t})+c_{2}\times r_{2}\times ({\mathrm{GB}}^{t}-x_{i}^{t}),$$

where *ω* is the inertia weight, *c*_1_ and *c*_2_ represent individual and social cognitive factors, respectively, and *r*_1_ and *r*_2_ are random numbers between [0, 1]. Assuming there are *N* populations, ${\mathrm{PB}}_{i}^{t}$ and ${\mathrm{GB}}^{t}$ represent the historical best positions found by the *i*-th and all *N* particles up to time *t*.

At time *t* + 1, *x* is updated according to the current position $x_{i}^{t}$ of the particle and the rate of change $v_{i}^{t+1}$ towards the next position, as shown in Equation (2) [16]:

(2) $$x_{i}^{t+1}=x_{i}^{t}+v_{i}^{t+1}.$$

## 3. The Proposed IA-DTPSO

In this section, we introduce five strategies to optimize PSO and propose an improved PSO (IA-DTPSO) method. The proposed IA-DTPSO is described in detail below.

### 3.1. Sobol Sequence Initialization

**Figure 1:**
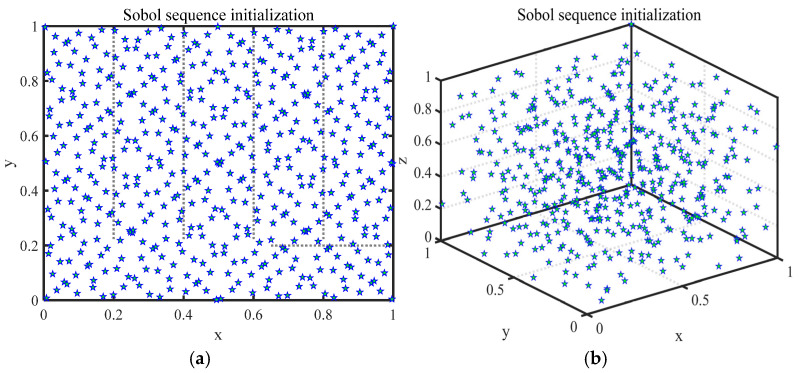
Population spatial distribution of Sobol sequence initialization. (**a**) Distribution in 2D space. (**b**) Distribution in 3D space.

The initial solutions’ distribution is an important prerequisite for affecting the convergence speed of MAs. A homogeneously spread initial population can effectively improve the search efficiency of MAs. Therefore, this article uses a Sobol sequence initialization [55] population instead of the random initialization scheme in PSO. The Sobol sequence is a low-variance sequence that uses a deterministic quasi-random number sequence instead of a pseudo-random number sequence to fill, as evenly as possible, points into a multidimensional hypercube, thereby generating wider coverage in the solution space. The initial population position generated by the Sobol sequence is shown in Equation (3) [55]:

(3) $$X_{i}=\mathrm{lb}+{\mathrm{Sobol}}_{i}\times (\mathrm{ub}-\mathrm{lb}),\;{\mathrm{Sobol}}_{i}\in [0,\;1],$$

where ***Sobol****_i_* denotes the *i*-th randomly generated number in the sequence. ***ub*** and ***lb*** denote the upper and lower bounds, respectively. The population spatial distribution of Sobol sequence initialization is shown in Figure 1.

### 3.2. Information Acquisition Strategy

The information acquisition strategy is to collect and acquire useful information through the key stages of information gathering, information filtering and evaluation, information analysis and organization.

#### 3.2.1. Information Gathering

Information gathering is a crucial step in gaining valuable feedback. Therefore, particles use various approaches and utilize a variety of channels to gather information, forming a more complete initial information system. This procedure can be expressed as [32]:

(4) $$x_{i}^{t+1}=x_{i}^{t}+\mu \times (x_{r_{1}}^{t}-x_{r_{2}}^{t}),$$

where $x_{r_{1}}^{t}$ and $x_{r_{2}}^{t}$ are two randomly generated particles at time *t*. *μ* is used as a random number between [−1, 1] to control the strength and direction of particle information collection. Generally speaking, collecting more information is not necessarily better. A large amount of information may lead to new candidate solutions exceeding the global optimum, thereby weakening the exploitation of the equation.

#### 3.2.2. Information Filtering and Evaluation

After the particles have collected the information, they need to quickly identify the relevant useful information, and this key mechanism can be expressed as

(5) $$\left\{\begin{array}{l} x_{i}^{t+1}=x_{i}^{t}-\sigma \times r_{3}\times (x_{r_{N}}^{t}-x_{i}^{t}),\;\mathrm{if}\;r_{3}<0.5,\\ x_{i}^{t+1}=x_{i}^{t}+\sigma \times r_{3}\times (x_{r_{N}}^{t}-x_{i}^{t}),\;\mathrm{else}, \end{array}\right.$$

where *r*_3_ is a random number between [0, 1], and *r_N_* is a particle randomly selected from *N* populations. σ is the error generated when subjective factors filter and evaluate information, defined by Equations (6)–(9) [32]:

(6) $$\sigma =\frac{\cos(\frac{\pi }{2}\times \sqrt{\left|\gamma \right|})}{\Omega },$$

(7) $$\Omega =2\times [3.468\times r_{4}\times (1-r_{5})\times \arccos(r_{6}\times 10^{4})],$$

(8) $$\gamma =\lambda +\sin\left({\left(\frac{\pi }{4}\right)}^{\frac{it}{T}}\right)+\frac{\log_{10}\left(\frac{it}{T}\right)}{8},$$

(9) $$\lambda =\cos(2\times r_{7}+1)\times (1-\frac{it}{T}),$$

where Ω is the subjective influencing factor, which serves as a quantitative indicator of particle subjectivity. It may make two extreme judgments on the information, thereby changing the result of information acquisition. Owing to changes in subjective states, the evaluation of different particles or the same particle at different time points may vary. Another key factor is γ, which characterizes the algorithm’s ability to self-adjust based on the information quality at different iteration stages. Among it, λ represents the information quality factor, which avoids the algorithm from neglecting the basic requirements of information quality due to excessive optimization iteration dynamics. Furthermore, *r_i_* (*i* = 4, 5, 6, 7) is a random number between [0, 1] in these equations.

**Figure 2:**
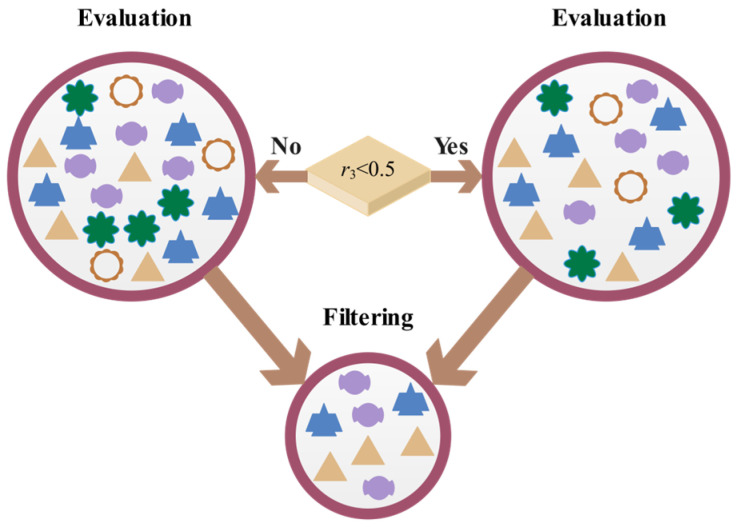
Schematic diagram of information filtering and evaluation.

Figure 2 shows a schematic diagram of information filtering and evaluation, from which it can be seen that particles exhibit adaptive adjustment behavior when evaluating different information, which not only effectively eliminates unconventional information but also significantly improves the overall quality of information.

#### 3.2.3. Information Analysis and Organization

**Figure 3:**
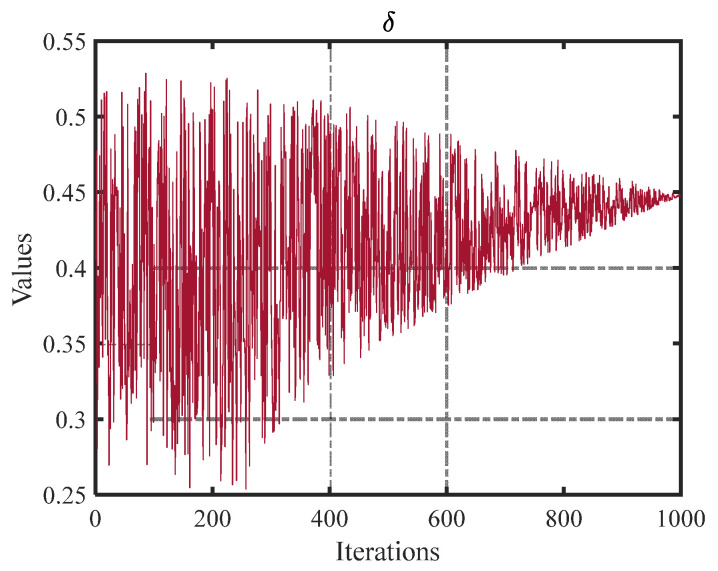
Trend plot of control factor δ.

After filtering out the information, particles need to seek out existing valuable information. They increase the likelihood of obtaining the optimal target information by converting the convertible information identified in the preceding stage into valuable information. This operation can be shown by Equations (10) to (11) [32]:

(10) $$\left\{\begin{array}{l} x_{i}^{t+1}={\mathrm{GB}}_{i}^{t}\times \cos(\frac{\pi }{2}\times \sqrt{\delta^{\frac{1}{3}}})-r_{8}\times (\frac{1}{N}\sum_{i=1}^{N}{\mathrm{GB}}_{i}^{t}-{\mathrm{GB}}_{i}^{t}),\;\mathrm{if}\;\lambda \geq 0.5,\\ x_{i}^{t+1}={\mathrm{GB}}_{i}^{t}\times \cos(\frac{\pi }{2}\times \sqrt{\delta^{\frac{1}{3}}})-0.8\times (r_{9}\times r_{10}\times \frac{1}{N}\sum_{i=1}^{N}{\mathrm{GB}}_{i}^{t}-(2\times r_{11}-1)\times {\mathrm{GB}}_{i}^{t}),\;\mathrm{else}, \end{array}\right.$$

(11) $$\delta =2^{\left(\sqrt{\left|\gamma \right|}-2\right)}.$$

where *r_i_* (*i* = 8, 9, 10, 11) is a random number between [0, 1]. δ indicates a controlling factor, the trend of which is shown in Figure 3.

**Figure 4:**
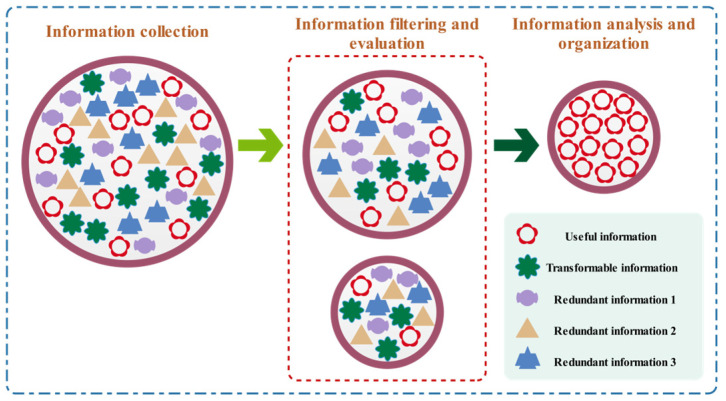
Schematic diagram of information acquisition strategy.

During this process, particles can optimize the depth and breadth of this stage dynamically, according to the quality of the information, thus increasing the target information body’s accuracy. In the next iteration, the novel information subject will totally substitute the previous information subject. Figure 4 depicts the entire framework of the information acquisition strategy.

### 3.3. SCC Method

SCC is a method used to evaluate the statistical dependence between two ranking sequences (here are two candidate solution positions) [56]. By measuring the consistency of the particle ranking differences between two candidate solutions, the statistical correlation between these two rankings can be evaluated. The expression of this method is shown in Equation (12) [56]:

(12) $$S_{C}=1-\frac{1}{D\times (D^{2}-1)}\times \sum_{i=1}^{N}(6z_{i}^{2}),$$

where *z_i_* = *m_i_* − *n_i_*, where *m_i_* and *n_i_* are the rankings of *N* populations in two sequences, respectively. When two sequences are completely identical, they are considered positively correlated. In this case, *m_i_* = *n_i_*. For each individual *i*, there is $\sum_{i}z_{i}^{2}=0$, so *S_C_* = 1. Similarly, when there is inconsistency between two sequences, it can be inferred that *S_C_* < 1.

In IA-DTPSO, the SCC’s calculation method shown in Equation (4) is used to measure the correlation between ***GB*** and each particle in each dimension, which determines the dimension that requires an inverse solution position update. The specific calculation formulas are shown in Equations (13)–(15) [56]:

(13) $$x_{i}^{t+1}(\tau )=\mathrm{ub}(\tau )+\mathrm{lb}(\tau )-x_{i}^{t}(\tau ),\tau \in \left\{j:z_{i}(j)>a\right\},$$

(14) $$z_{i}(j)=\left|\mathrm{GB}(j)-x_{i}(j)\right|,$$

(15) $$a=2-\frac{2\times it}{T},$$

where *j* is the *j*-th dimension on the *D*-dimensional problem. This targeted non-complete reverse operation helps the algorithm improve computational accuracy while maintaining its fast convergence.

### 3.4. Tangent Flight Strategy

Due to the fact that there is only one update method in PSO that calculates the next position based on the rate of change, this single-search method often carries the risk of convergence stagnation. Therefore, based on the PSO update method, this section utilizes the tangent flight strategy to compensate for this deficiency. The updated formula obtained by combining Equations (1) and (2) and introducing the tangent flight strategy is shown in Equation (16) [43]:

(16) $$\left\{\begin{array}{l} x_{i}^{t+1}=x_{i}^{t}+v_{i}^{t+1},\;\mathrm{if}\;r_{12}<0.5,\\ x_{i}^{t+1}=x_{i}^{t}+step\times \tan(\theta ),\;\mathrm{else},\; \end{array}\right.$$

where *r*_12_ is a random number between [0, 1]. In tangent flight, all motion equations are controlled by a global step, which takes the form of *step* × tan(*θ*), and *θ* is a random number between $\left[0,\;\frac{\pi }{2}\right)$. *step* is the move’s size, and its calculation formula is

(17) $$step=\mathrm{sign}(r_{13}-0.5)\times \mathrm{norm}({\mathrm{GB}}_{i}^{t})\times \log_{10}(1+\frac{10\times D\times N}{it\times T}),$$

where *r*_13_ is a random number between [0, 1], the sign controls the direction of ENE, and the norm is a Euclidean norm.

**Figure 5:**
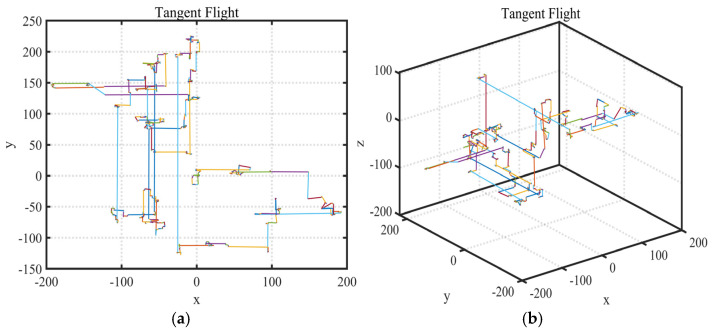
Schematic diagram of tangent flight random walk. (**a**) Distribution in 2D space. (**b**) Distribution in 3D space.

As shown in Figure 5a,b, the stride interval generated by tangent flight is large and the stride randomness is small, which keeps the search distance stable during the iteration process and greatly shortens the optimization iteration cycle of the algorithm. In addition, particles can obtain more information in this large step frequency search to get rid of local optima’s constraints.

### 3.5. Dimension Learning Strategy

For particles in PSO, they can only passively be limited by the radiation of ***PB*** and ***GB***, and cannot extract more effective information from other particles. In the introduced dimension learning strategy, particles can learn based on the behavior of their neighbors. Calculate the radius between the particle and other candidate particles based on the Euclidean distance [57]:

(18) $$R_{i}^{t}=\left\|x_{i}^{t}-x_{i}^{t+1}\right\|,$$

The neighborhood of $x_{i}^{t}$ can be expressed as:

(19) $$U_{i}^{t}=\left\{\left.x_{k}^{t}\right|\Delta_{i}(x_{i}^{t},x_{k}^{t})\leq \left.R_{i}^{t}\right|,\;x_{k}^{t}\in N\right\}\;,$$

where $\Delta_{i}$ is the Euclidean distance between $x_{i}^{t}$ and $x_{k}^{t}$. Once the neighborhood of $x_{i}^{t}$ is constructed, a neighbor particle can be randomly selected from the *j*-th dimensional neighborhood for updating using Equation (20):

(20) $$x_{i}^{t+1}(j)=x_{i}^{t}(j)+r_{14}\times (x_{u}^{t}(j)-x_{r}^{t}(j)),$$

where *r*_14_ is a random number between [0, 1], $x_{u}^{t}(j)$ is a randomly selected neighbor from the neighborhood $U_{i}^{t}$, and $x_{r}^{t}(j)$ is a randomly selected particle from *N* populations.

Dimension learning strategy increases the algorithm’s exploration capacity and ability to retain population diversity by increasing the interaction between particles and their neighbors and introducing other randomly selected particles from the population.

**Figure 6:**
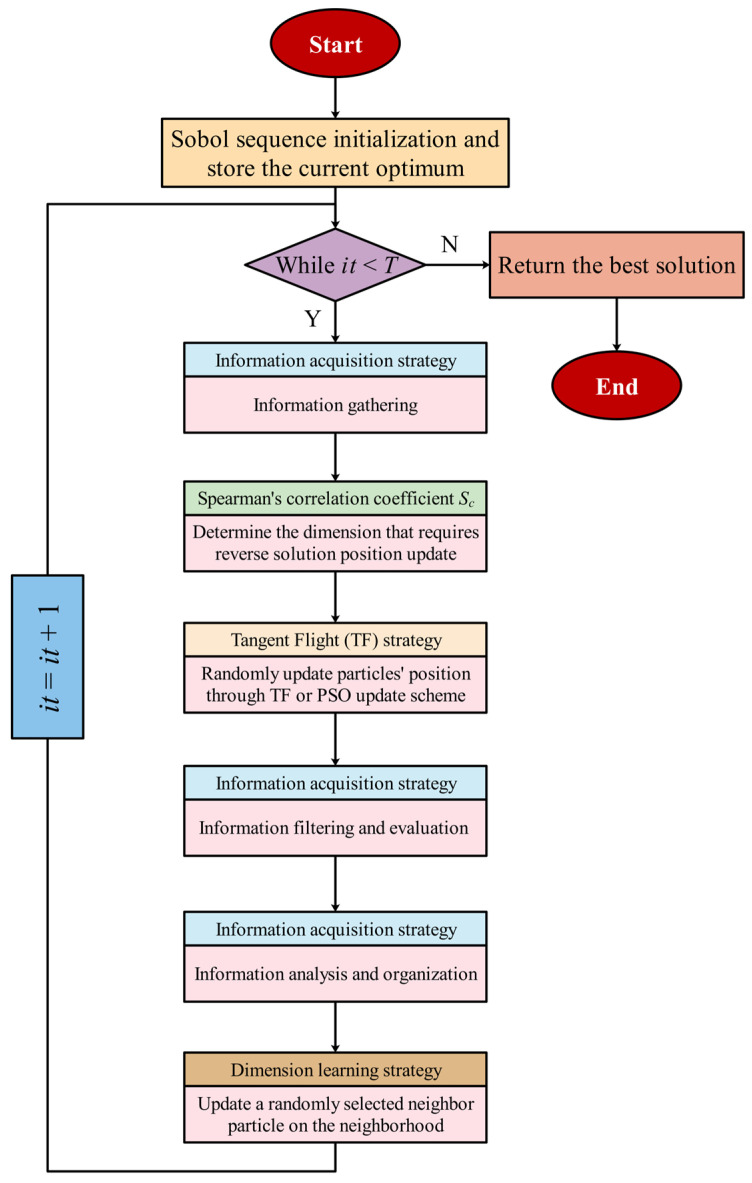
The flowchart of the proposed IA-DTPSO.

In order to present the structure and process of IA-DTPSO more intuitively, Algorithm 1 provides IA-DTPSO’s pseudo-code and draws IA-DTPSO’s flowchart as mirrored in Figure 6.
**Algorithm 1:** IA-DTPSO’s pseudo-codeStart IA-DTPSO Input: Particles’ number (*N*) and iterations (*T*) Output: The optimum 1:  Use Equation (3) for Sobol sequence initialization and store the current optimum 2: While (*it < T*) Do 3:    For *i* = 1 to *N* Do 4:   Use Equation (4) to form the initial information system 5:    End For 6:    Update the parameter *a* using Equation (15) 7:    Calculate the Spearman’s correlation coefficient *S_c_* using Equations (12) and (14) 8:    For *i* = 1 to *N* Do 9:   For *j* = 1 to *D* Do 10:    If *S_c_* <= 0 11:     For $\tau \in \left\{j:z_{i}(j)>a\right\}$ 12:      Use Equation (13) to determine the dimension that requires reverse solution position update 13:     End For 14:    End If 15:   End For 16:    End For 17:    For *i* = 1 to *N* Do 18:   Calculate the movement size *step* using Equation (15) 19:    Use Equation (16) for the tangent flight or PSO update scheme to randomly update particles’ position 20:    End For 21:    For *i* = 1 to *N* Do 22: Exploration 23:   Update relevant parameters using Equations (6)–(9) 24:   Use Equation (5) for information filtering and evaluation process 25: End 26: Exploitation 27:   Update parameter δ using Equation (11) 28:   Use Equation (10) for information analysis and organization 29: End 30:    End For 31:    For *i* = 1 to *N* Do 32:   Update radius $R_{i}^{t}$ using Equation (18) 33:   Construct the neighborhood $x_{i}^{t}$ using Equation (19) 34:   For *j* = 1 to *D* Do 35:    Update a randomly selected neighbor particle on the neighborhood using Equation (20) 36:   End For 37:    End For 38:    Compute fitness values and store the current optimum 39:    *it* = *it* + 1 40: End While 41: Output the optimumEnd IA-DTPSO

### 3.6. Time Complexity Analysis of IA-DTPSO

In this section, we discuss the time complexity of IA-DTPSO. The time complexity of IA-DTPSO mainly depends on four parts: Sobel sequence initialization *O*(*N* × *D*), fitness evaluation *O*(5 × *N*), fitness ranking *O*(*N* × log*N*), and update *O*(5 × *N* × *D*). Therefore, the time complexity of IA-DTPSO is as follows:

(21) O(IA-DTPSO)=O(N×D+N×T×(5+logN+5×D)).

## 4. Experimental Results and Discussion

### 4.1. Experimental Design and Parameter Setting

In this section, we test IA-DTPSO with 11 different types of comparison algorithms on the 10 and 20 dimensions of CEC2022 [23]. This test set provides a train of challenging test functions, as shown in Algorithm 1. By inputting variables *N* and *T*, continuous position updates and iterations are carried out until the optimum is output. The entire process reflects the solving performance of IA-DTPSO on these functions. Therefore, CEC2022 is an effective tool for fair comparison between different MAs. Comparative algorithms can be categorized into the following two types:

(1) New MAs proposed in recent years: RUNge Kutta Optimizer (RUN) [58], Northern Goshawk Optimization (NGO) [19], Nutcracker Optimization Algorithm (NOA) [21], Genghis Khan Shark Optimizer (GKSO) [23], and IVY Algorithm (IVYA) [59].
(2) PSO [17] and its various improved versions: Elite Archives-driven PSO (EAPSO) [60], Gaussian Quantum-behaved PSO (G-QPSO) [61], Hybrid algorithm based on Jellyfish Search PSO (HJSPSO) [62], single-objective variant PSO (PSO-sono) [63], and Multi-strategy PSO incorporating Snow Ablation Optimizer (SAO-MPSO) [64].

Table 1 displays the parameter settings for each MA. To avoid the influence of unexpected factors on the experiment, the *runs* are set to 20, which means that all MAs are independently run 20 times on each test function. Meanwhile, set *N* to 100 and *T* to 1000. Evaluate the optimization results through six evaluation metrics: Best, Worst, Mean, Wilcoxon Rank Sum Test (WRST), Friedman Test (FT), and Rank. In addition, optimization results are evaluated through three error indicators: Standard deviation (Std), Root Mean Square Error (RMSE), and relative error (*δ*). All tests are conducted according to the equipment specifications displayed in Table 2.

### 4.2. ENE Behavior Analysis

In order to further confirm that the improvement strategy proposed in this study is promising and effective in solving potential problems, this section discusses the trend of ENE rate changes of IA-DTPSO on CEC2022. The relevant formulas are as follows [64]:

(22) $$Div_{i}=\frac{1}{N}\sum_{i=1}^{N}\hat{M}(x(j))-x_{i}(j),$$

(23) $$Div=\frac{1}{D}\sum_{i=1}^{D}Div_{i},$$

(24) $$E_{1}\%=\frac{Div}{Div_{\max}}\times 100\%,$$

(25) $$E_{2}\%=\frac{\left|Div-Div_{\max}\right|}{Div_{\max}}\times 100\%,$$

where $\hat{M}(x(j))$ denotes all particles’ median on the *j*-th dimension. *Div*_max_ indicates the maximum diversity. *E*_1_% and *E*_2_% mean exploration rate and exploitation rate, respectively.

**Figure 7:**
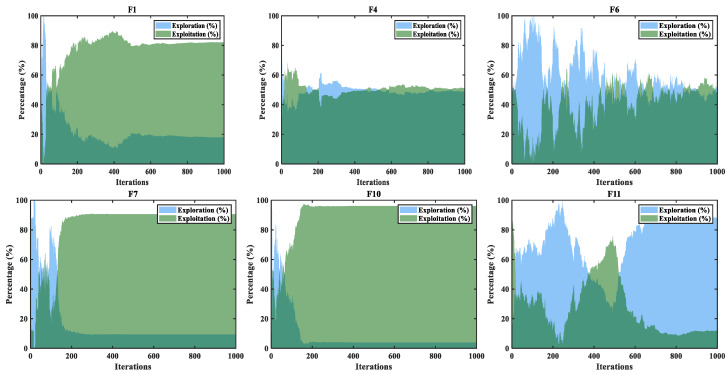
ENE trends of IA-DTPSO on CEC 2022 partial test functions.

The two intersecting nonlinear curves shown in Figure 7 represent the ENE change rate of IA-DTPSO on the CEC2022 partial function. From these graphs, it can be seen that ENE rapidly approaches the intersection point at the beginning of iteration, which is attributed to the fact that the initial population under Sobol sequence initialization can effectively improve the search efficiency of particles. Subsequently, ENE reaches the first equilibrium point, where the two are intertwined and the ENE rate is 50%. This is due to the fact that the updated equations of Equations (5) and (9) in the information acquisition strategy effectively balance ENE. As shown in F1, F7, and F10 in Figure 7, after the first intersection of ENE, IA-DTPSO quickly transitioned from exploration to exploitation, focusing on local exploitation and completing the entire search in subsequent iterations. This is due to the targeted non-complete reverse operation of SCC, which helps IA-DTPSO improve convergence accuracy. However, ENE may experience fluctuations and even multiple intersections on certain functions. As shown in F6, ENE intersects several times at 50% and eventually stabilizes. This is because in tangent flight, the random step frequency and size generated by particles can obtain more information, and this random execution of exploration or exploitation operations can effectively break free from the constraints of local optima. The dimension learning strategy enhances the algorithm’s exploration ability and ability to maintain population diversity by increasing the interaction between particles and their neighbors. Specifically, as shown in F11, after 500 iterations, the proportion of exploration continues to increase, indicating that IA-DTPSO is still searching for the global optimum. In summary, the proposed strategies play their respective roles in solving CEC2022 and jointly promote the convergence of IA-DTPSO towards the theoretical optimum.

### 4.3. Experimental Results and Analysis

Table 3 displays statistical results of IA-DTPSO and other MAs on 10-dimensional CEC2022, with the optimal data highlighted in bold. In addition, the Theoretical Optimal (TO) values for F1–F12 in CEC2022 are 300, 400, 600, 800, 900, 1800, 2000, 2200, 2300, 2400, 2600, and 2700, respectively. All values that reach theoretical optimum in experimental results of this section are replaced by “TO”. For an algorithm, the more times it reaches TO, the better its performance. Firstly, IA-DTPSO achieves smaller values on 7 out of 12 test functions, accounting for 58.33% of CEC2022. Secondly, IA-DTPSO performs particularly well on uni-modal functions (F1), hybrid functions (F6–F8), and composition functions (F9–F12), all of which have achieved at least the top 2 rankings. Finally, according to the final ranking results, IA-DTPSO has a mean rank of 1.833 and a mean FT of 2.681, both leading the other comparison MAs. This denotes that IA-DTPSO has a great ability to solve complex optimization problems, and a smaller FT also means that IA-DTPSO has better stability. If FT is designed for repeated testing, then WRST is designed to test the pairing between two groups. Table 3 presents the WRST results under the condition of significance level α = 0.05. The symbol “-” denotes comparison algorithms’ number that are inferior to IA-DTPSO; “+” is the quantity of opposite effects to “-”; “=“ represents the number of algorithms with similar performance compared to IA-DTPSO. The final functions’ numbers that are superior/similar/inferior to IA-DTPSO for each comparison algorithm are 2/0/10, 0/1/11, 4/3/5, 0/0/12, 0/1/11, 0/0/12, 1/2/9, 0/0/12, 3/2/7, 0/0/12, and 0/2/10, respectively. The WRST results show that NOA, IVYA, G-QPSO, and PSO-sono obtain the same test data, and these algorithms do not outperform IA-DTPSO on any of the functions. RUN and GKSO also obtain the same test data, which only show similar performance to IA-DTPSO on one function and inferior to IA-DTPSO on the other functions. Although NGO and HJSPSO, ranked second and third respectively, outperform IA-DTPSO in some functions, they are inferior to IA-DTPSO in more functions. Therefore, it can be said that NGO and HJSPSO also have certain competitiveness. In addition, PSO ranked seventh, which is inferior to IA-DTPSO on 10 functions, indicating that IA-DTPSO has significantly improved its optimization ability compared to PSO. However, PSO still outperforms IA-DTPSO on two functions, and further improvement of IA-DTPSO’s performance on these functions can be considered in the future.

To further compare the discrepancy in performance between IA-DTPSO and various MAs, Table 4 provides the error data for IA-DTPSO and other algorithms in solving the 10-dimensional CEC2022. Based on the relevant data in Table 4, the Std values of PSO are relatively high on F1 and F6, indicating that PSO exhibits instability on different test functions. Meanwhile, the RMSE and *δ* values of PSO on F6 are also high, indicating that PSO has low accuracy in complex problems and inconsistent performance in different runs. In addition to PSO, NOA, IVYA, and PSO-sono also perform poorly on complex problems, with low stability and accuracy. EAPSO and NGO perform well on simple issues but are still slightly inferior to IA-DTPSO on complex issues. IA-DTPSO gains relatively small RMSE and *δ* values on most functions, suggesting that IA-DTPSO has high accuracy to assure that more experimental results are close to the TO solution.

**Figure 8:**
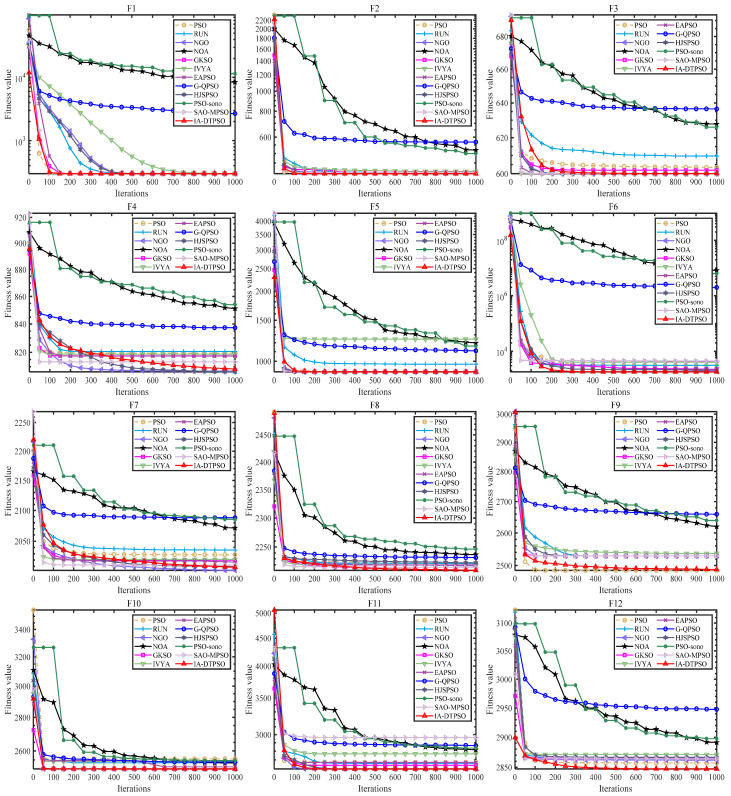
Convergence curves of IA-DTPSO and other MAs for addressing 10-dimensional CEC2022.

**Figure 9:**
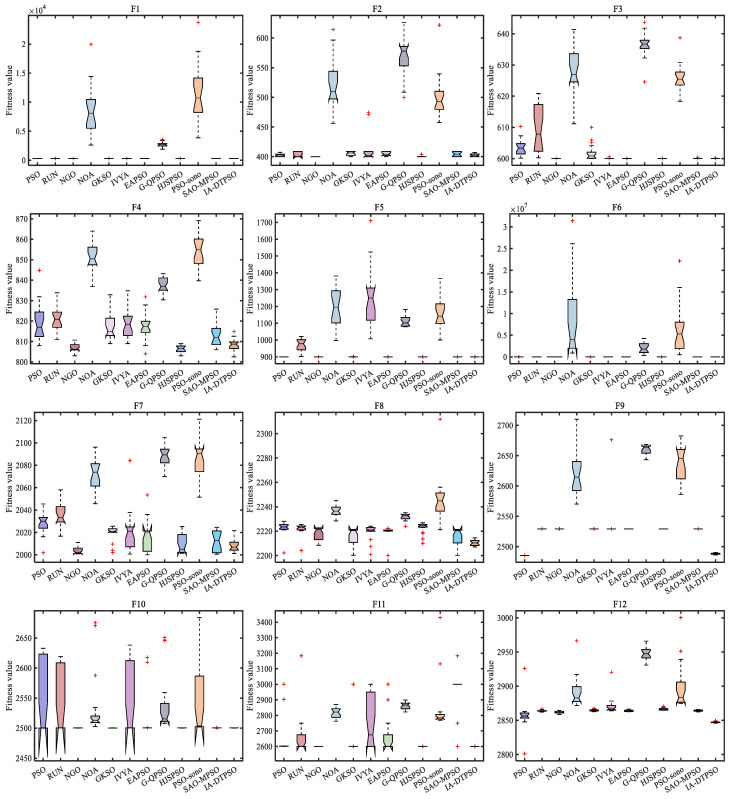
Box plots of IA-DTPSO and other MAs for solving 10-dimensional CEC2022.

Figure 8 mirrors IA-DTPSO’s convergence curves and other MAs on 12 functions. IA-DTPSO quickly converges to the global optimum in the early stage and finds the TO solution around 100 iterations. This is due to the Sobol sequence initialization generating a good initial solution for the particle population, which gives IA-DTPSO extraordinary optimization ability. In addition, IA-DTPSO still has a downward convergence trend after 1000 iterations when solving the F4 function, indicating that IA-DTPSO still has the capacity to find the global optimum. This is also due to the information acquisition strategy that maintains the diversity between different particles, resulting in a steady increase in population diversity. Based on the box plots shown in Figure 9, IA-DTPSO’s box shape is relatively narrow and positioned downwards, indicating that IA-DTPSO has good robustness and high accuracy.

Table 5 presents the statistical results of IA-DTPSO and other MAs on 20-dimensional CEC2022. Firstly, IA-DTPSO achieves smaller values on 5 out of the 12 test functions, accounting for 41.67% of CEC2022. Secondly, IA-DTPSO performs particularly well on uni-modal functions (F1) and hybrid functions (F6–F8), both ranking at least in the top two. Finally, according to the final ranking results, IA-DTPSO has a mean rank of 2.417 and a mean FT of 3.242, both leading the other comparison algorithms. This indicates that with the increase in problem dimensions, IA-DTPSO still has a positive optimization ability. Meanwhile, Table 5 presents the WRST results of IA-DTPSO and other MAs. The number of functions obtained for each comparison MA that are superior/similar/inferior to IA-DTPSO is 1/1/10, 0/2/10, 3/2/7, 0/0/12, 0/2/10, 1/0/11, 3/4/5, 0/0/12, 4/1/7, 0/0/12, and 4/3/5, respectively. From the WRST results of this group, NOA, G-QPSO, and PSO-sono obtain the same test data, and these algorithms do not outperform IA-DTPSO on any of the functions. Similar to the test results on the 10-dimensional CEC2022, RUN and GKSO also obtain the same settlement data. They only perform similarly to IA-DTPSO on one function and are inferior to IA-DTPSO on the other functions. In addition, PSO ranked eighth and only outperformed IA-DTPSO on one function and is inferior to IA-DTPSO on ten functions, indicating that IA-DTPSO has significantly improved its optimization level compared to PSO. It is worth mentioning that HJSPSO and SAO-MPSO, ranked fourth and fifth, respectively, have a higher number of functions than IA-DTPSO, NGO, and EAPSO, ranked second and third, respectively. Combined with the mean rank outcomes, there is no appreciable discrepancy in the performance of these MAs.

Table 6 provides error data for IA-DTPSO and other algorithms in solving 20-dimensional CEC2022. Based on the relevant data of the best and worst values in Table 5, IA-DTPSO has smaller Std values on most functions. Combined with the relevant data of Mean in Table 5, IA-DTPSO also gains smaller RMSE and *δ* values on most functions. Thus, IA-DTPSO has high accuracy and always approaches the TO solution with minimal error, making it the algorithm with the best overall performance. In addition, EAPSO and SAO-MPSO perform well on simple problems, but are slightly inferior to IA-DTPSO on complex problems. It is worth mentioning that PSO, NOA, IVYA, and PSO-sono still perform poorly in high-dimensional complex problems, manifested in low stability and accuracy.

**Figure 10:**
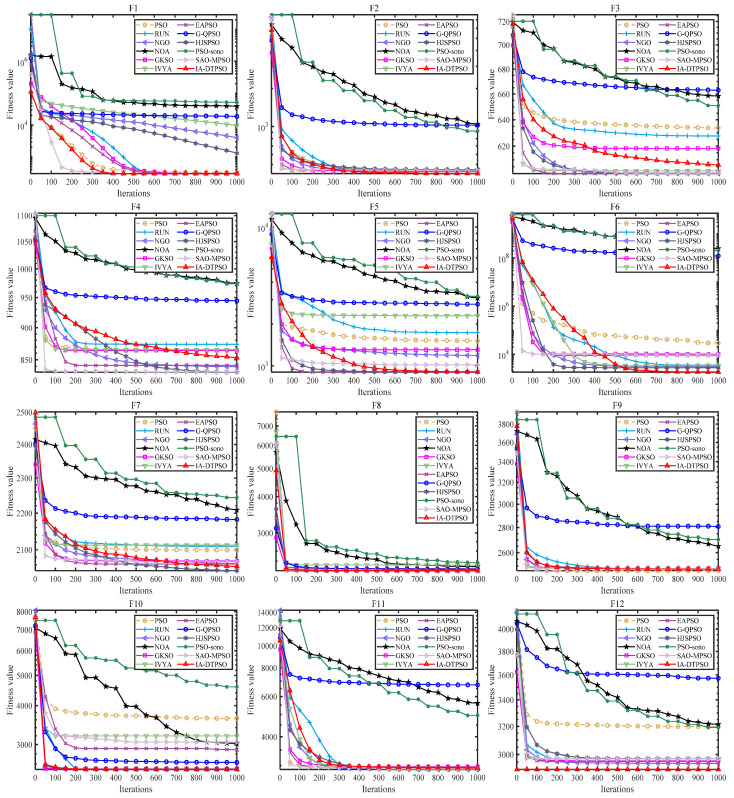
Convergence curves of IA-DTPSO and other MAs for solving 20-dimensional CEC2022.

**Figure 11:**
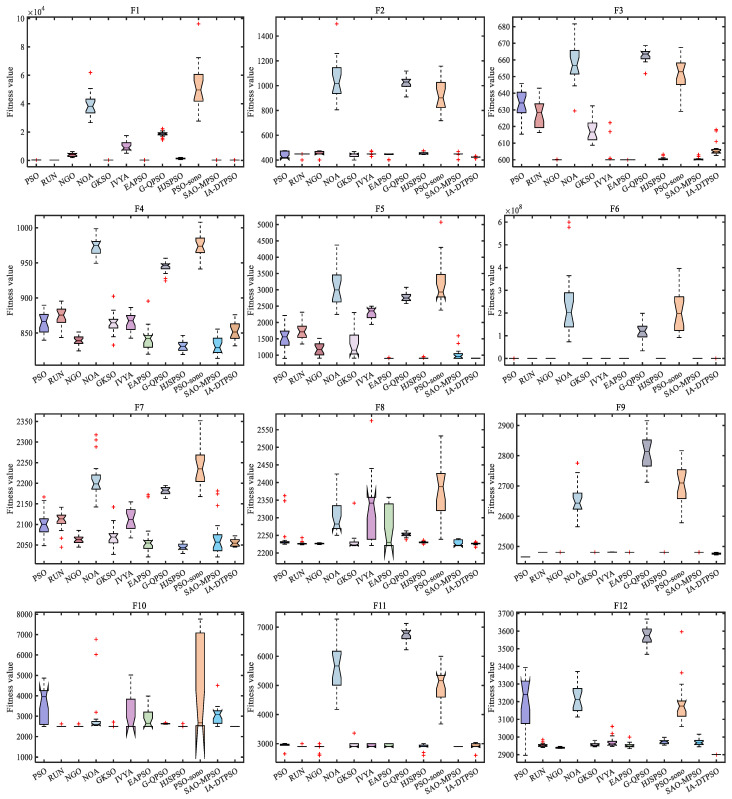
Box plots of IA-DTPSO and other MAs for solving 20-dimensional CEC2022.

Figure 10 mirrors the convergence curves of IA-DTPSO and other MAs on a 20-dimensional CEC2022. IA-DTPSO has a faster convergence rate on most functions, and its convergence curve can reach a lower landing point within a limited number of iterations. In addition, IA-DTPSO still has a downward convergence trend after iteration termination when solving F3, F4, and F7 functions, indicating that IA-DTPSO still has the capacity to find the global optimum. Figure 11 mirrors the box plots of IA-DTPSO and other MAs on a 20-dimensional CEC2022. IA-DTPSO’s box shape is relatively narrow and positioned downwards, indicating that IA-DTPSO has good robustness and high accuracy.

**Figure 12:**
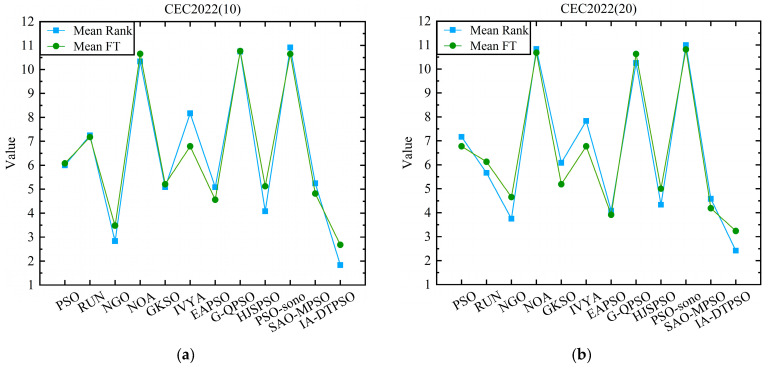
Comparative line graphs of mean rank and mean FT for each MA on different dimensions of CEC2022. (**a**) Comparison results on 10-dimensional CEC2022. (**b**) Comparison results on 20-dimensional CEC2022.

**Figure 13:**
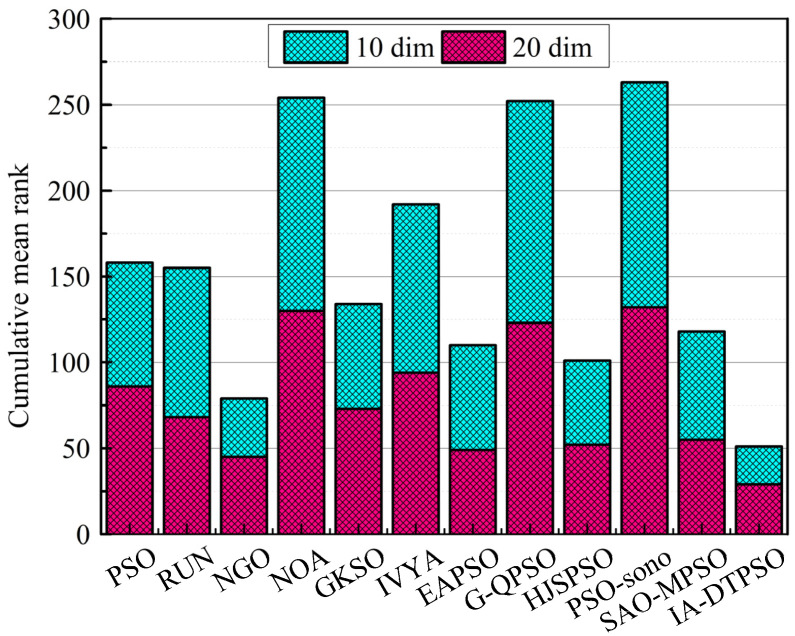
Cumulative rank sum of IA-DTPSO and other MAs on different dimensions.

Figure 12 shows a line graph comparison of the mean rank and mean FT of IA-DTPSO and other algorithms in different dimensions of CEC2022. Among them, (a) is the comparison result in 10 dimensions, and (b) is the comparison result in 20 dimensions. From the two sub-graphs in Figure 12, IA-DTPSO has the smallest mean rank and mean FT in both dimensions, which is due to the stable performance of IA-DTPSO in both dimensions under the influence of the dimension learning strategy. In addition, owing to discrepancies in statistical approaches, the mean FT and mean rank of each algorithm also vary slightly. The smaller this difference, the better the stability of the algorithm’s operation. Finally, Figure 13 mirrors the stacked rank and bar chart of IA-DTPSO and other algorithms in different dimensions and finds that IA-DTPSO obtains the lowest cumulative column height. In summary, the comprehensive performance of IA-DTPSO is obviously better than the other compared MAs.

## 5. Simulation and Prediction of TUWRs in China Based on IA-DTPSO and TDGM(1,1,*r*,*ξ*,*Csz*)

We verified the superiority of the proposed IA-DTPSO on the test set. In this section, we use IA-DTPSO to optimize TDGM (1,1,*r*,*ξ*,*Csz*) and apply the optimized TDGM (1,1,*r*,*ξ*,*Csz*) model to simulate and predict TUWRs’ situation in China.

### 5.1. TDGM(1,1,r,ξ,Csz)

**Definition** **1.** *Let a set of data sequences* $X^{\left(0\right)}=(x^{\left(0\right)}(1),x^{\left(0\right)}(2),\ldots ,x^{\left(0\right)}(n))$, $X^{\left(r\right)}$ *is a one-time accumulation sequence of *$X^{\left(0\right)}$*, as shown in Equation (26):*

(26) $$X^{\left(r\right)}=(x^{\left(r\right)}(1),x^{\left(r\right)}(2),\ldots ,x^{\left(r\right)}(n)),\;r\in R^{+},$$

*where the calculation formula for* $X^{\left(r\right)}(n)$ *is shown in Equation (27):*

(27) $$X^{\left(r\right)}(n)=\sum_{i=1}^{n}\frac{\Gamma (r+n-i)}{\Gamma (n-i+1)\Gamma (r)}x^{\left(0\right)}(i),$$

**Figure 14:**
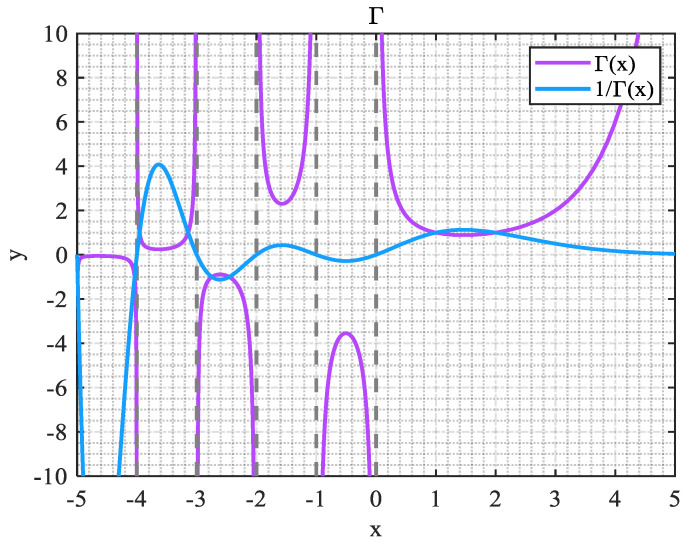
Γ Function.

*where* Γ* function is utilized to optimize the space of order r in the model. Figure 14 shows the graph of Γ function.*

It is not difficult to derive the expression for the inverse first-order accumulation sequence $X^{\left(-r\right)}$ of $X^{\left(0\right)}$ from Definition 1, which will not be repeated here.

**Definition** **2.** $Z^{\left(r\right)}$ *is the average sequence generated by consecutive neighboring neighbors of* $X^{\left(r\right)}$*, as shown in Equation (28):*

(28) $$Z^{\left(r\right)}=(z^{\left(r\right)}(2),z^{\left(r\right)}(3),\ldots ,z^{\left(r\right)}(n)),\;r\in R^{+},$$

*where the calculation method of* $z^{\left(r\right)}(n)$ *is shown in Equation (29):*

(29) $$z^{\left(r\right)}(n)=\xi \times x^{\left(r\right)}(n)+(1-\xi )\times x^{\left(1\right)}(n-1),$$

**Definition** **3.** *If *$X^{\left(0\right)}$, $X^{\left(r\right)}$*, and* $Z^{\left(r\right)}$ *have the same definitions as above, then there are*

(30) $$x^{\left(r-1\right)}(k)+az^{\left(r\right)}(k)=kb+c,k=1,2,\ldots ,n,$$

*Equation (30) is denoted as TDGM (1,1,r,ξ,Csz).*

**Theorem** **1.** *Let* $\hat{C}={\left(a,b,c\right)}^{T}$ *be computed as shown in Equation (31):*

(31) $$\hat{C}={\left(a,b,c\right)}^{T}={\left(B^{T}B\right)}^{-1}B^{T}Y,$$

*where **Y** and **B** are matrices (n − 1) × 1 and (n − 1) × 3, respectively, expressed as:*

(32) $$Y=\left[\begin{array}{c} x^{\left(r-1\right)}(2)\\ x^{\left(r-1\right)}(3)\\ \vdots \\ x^{\left(r-1\right)}(n) \end{array}\right],B=\left[\begin{array}{ccc} -z^{\left(r\right)}(2) & 2 & 1\\ -z^{\left(r\right)}(3) & 3 & 1\\ \vdots & \vdots & \vdots \\ -z^{\left(r\right)}(n) & n & 1 \end{array}\right].$$

**Theorem** **2.** *The r-order time response function of TDGM (1,1,r,ξ,Csz) is*

(33) $${\hat{x}}^{\left(r\right)}(k)=Csz\times \alpha_{2}^{k-1}+\sum_{g=0}^{k-2}[(k-g)\times \beta_{2}+\gamma_{2}]\times \alpha_{2}^{g},$$

*where *$\alpha_{2}=\frac{1-a\times (1-\xi )}{1+\xi \times a},\beta_{2}=\frac{b}{1+\xi \times a},\gamma_{2}=\frac{c}{1+\xi \times a},$ *and*

(34) $${\hat{x}}^{\left(0\right)}(k)={\left({\hat{x}}^{\left(r\right)}\right)}^{\left(-r\right)}(k)=\sum_{i=0}^{k-1}{\left(-1\right)}^{i}\frac{\Gamma (r+1)}{\Gamma (i+1)\Gamma (r-i+1)}{\hat{x}}^{\left(r\right)}(k-i).$$

The proof process of TDGM (1,1,*r*,*ξ*,*Csz*) is described in reference [65].

### 5.2. Investigation Data Analysis

TUWRs refer to the surface and underground water production formed by urban precipitation, which is the sum of surface runoff and precipitation infiltration recharge [66]. China is a country with abundant water resources, but also a country with scarce and unevenly distributed water resources [67]. With the acceleration of urbanization, people’s demand for water resources has sharply increased. In order to improve prediction accuracy and water resource utilization efficiency, this section selects the TUWRs in China from 2004 to 2023 for simulation and prediction. Table 7 presents the total urban water resources data in China over the past 20 years, sourced from the National Bureau of Statistics (https://data.stats.gov.cn/ access on 20 March 2025). As an important input data for this method, 75% of the data (2004–2018) will be used for the training set and 25% (2019–2023) for the test set.

**Figure 15:**
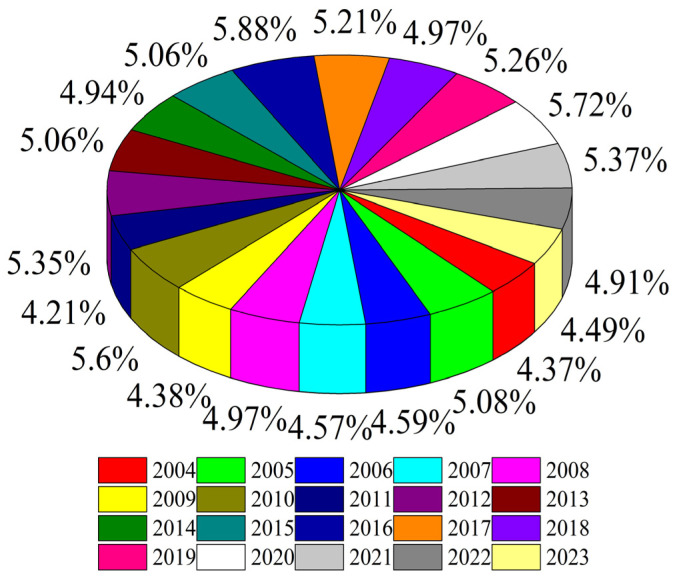
Distribution of the proportion of TUWRs in China from 2004 to 2023.

**Figure 16:**
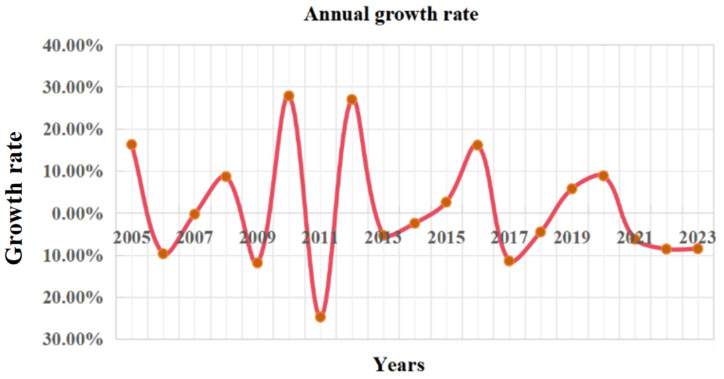
Growth rate of total urban annual water resources in China.

Figure 15 shows the distribution of the proportion of TUWRs in China from 2004 to 2023. It can be seen that China’s TUWRs do not increase linearly over time, and their annual TUWRs are influenced by actual social conditions. Figure 16 shows the growth rate of TUWRs in China, from which it can be seen that the total urban water resources growth rate was the most significant from 2009 to 2013, while the growth rate in the past two years has been relatively slow.

### 5.3. Model Evaluation Criteria

This study uses four error evaluation metrics to gauge the predictive performance of TDGM (1,1,*r*,*ξ*,*Csz*), including Absolute Percentage Error (APE), Mean Absolute Percentage Error (MAPE), simulation MAPE (MAPE*_simulation_*), and prediction MAPE (MAPE*_prediction_*). The descriptions of these indicators are shown in Equations (35)–(38) [68]:

(35) $$\mathrm{APE}=\frac{\left|\hat{x}(k)-x(k)\right|}{x(k)}\times 100\%,$$

(36) $${\mathrm{MAPE}}_{simulation}=\frac{1}{g}\sum_{i=1}^{g}\frac{\left|x(k)-\hat{x}(k)\right|}{x(k)}\times 100\%,$$

(37) $${\mathrm{MAPE}}_{prediction}=\frac{1}{n-g}\sum_{i=g+1}^{n}\frac{\left|x(k)-\hat{x}(k)\right|}{x(k)}\times 100\%,$$

(38) $$\mathrm{MAPE}=\frac{g\times {\mathrm{MAPE}}_{simulation}+(n-g)\times {\mathrm{MAPE}}_{prediction}}{n},$$

where $\hat{x}(k)$ and x(k) are the fitted value and the raw data, respectively.

### 5.4. IA-DTPSO and Other Algorithms for Parameter Optimization and Prediction of TDGM (1,1,r,ξ,Csz)

This section uses IA-DTPSO, PSO [17], GKSO [23], IVYA [59], EAPSO [60], HJSPSO [62], PSO-sono [63], and SAO-MPSO [64] to optimize TDGM (1,1,*r*,*ξ*,*Csz*) and applies them to simulate and predict TUWRs in China. Table 8 shows the statistical results of IA-DTPSO and other MAs for solving TUWRs in China. Among them, SimD represents simulated data, and ResE represents residuals. It is not difficult to see from Table 8 that all data except for the true values given over these years are output data. Among them, the MAPE*_simulation_* (%) and MAPE*_prediction_* (%) of IA-DTPSO on the training and testing sets are 5.6366 and 6.8041, respectively, and the total MAPE (%) is 5.9439. It can be seen that the three performance indicators of IA-DTPSO have achieved the minimum values compared with the other seven MAs. Furthermore, the MAPE*_simulation_* (%) and MAPE*_prediction_* (%) of PSO on the training and testing sets are 6.6432 and 7.2254, respectively, and the total MAPE (%) is 6.7964. Obviously, IA-DTPSO has significantly reduced the error in predicting TUWRs in China compared to PSO. Table 9 shows the parameters calculated by IA-DTPSO and other algorithms after optimizing the TDGM (1,1,*r*,*ξ*,*Csz*). Finally, Table 10 displays the forecast results of TUWRs in China in the next five years. TUWRs in China will reach 3368.846 hundred million cubic meters in 2028, which is the year with the highest total water resources since 2004. How to reasonably utilize and manage these huge water resources will be a challenge in the future.

### 5.5. Four Models for Simulating and Predicting TUWRs in China

In this section, the IA-DTPSO optimized TDGM (1,1,*r*,*ξ*,*Csz*) and existing GM (1,1) [69], DGM (1,1) [70], and NGBM (1,1) [71] models are used to simulate and predict TUWRs in China. The statistical results are shown in Table 11. From the output data in the table, it can be seen that the TDGM (1,1,*r*,*ξ*,*Csz*) model optimized by IA-DTPSO (referred to as ID_T) has a MAPE*_simulation_* (%) and a MAPE*_prediction_* (%) of 5.6366 and 6.8041 on the training and testing sets, respectively, and a total MAPE (%) of 5.9439. Its three performance indicators have reached the minimum values among the four compared models, further indicating the superiority of IA-DTPSO. Table 12 presents the forecast results of TUWRs in China for the next five years under four different models. It can be seen from the table that the GM (1,1), DGM (1,1), and NGBM (1,1) models predict a relatively flat growth trend in TUWRs for the next five years, while the TDGM (1,1,*r*,*ξ*,*Csz*) model optimized by IA-DTPSO shows a large fluctuation in the forecast results, which is closely related to the iteration and randomness of meta-heuristic methods and can better reflect the more fitting forecast data.

## 6. Conclusions and Future Prospects

This study proposes a multi-strategy improved PSO (IA-DTPSO). Firstly, Sobol sequences are introduced to produce a wider coverage of the initial particles. Secondly, an update mechanism based on information acquisition methods is established, which applies three different types of information processing operations to different stages. Among them, information gathering is the preparatory stage for particles to obtain useful information, and the remaining two methods correspond to the ENE stage of algorithms. This method improves the overall quality of information obtained by particles. Then, the SCC is introduced to gauge the correlation between ***GB*** and particles in each dimension, determining the dimensions that require reverse solution position updates, ensuring that the algorithm improves computational accuracy without sacrificing convergence speed. In addition, the use of tangent flight strategy combined with the original update method of PSO prevents the algorithm from falling into convergence stagnation. Finally, the introduced dimension learning strategy increases the interactivity between particles, enhances the overall particle vitality, and sustains the population diversity.

In the experimental section, the changing trend of the ENE rate of IA-DTPSO on CEC2022 was first discussed, further confirming the promising improvement strategy proposed in this study in solving potential problems. Then, by comparing IA-DTPSO with 11 other algorithms on different dimensions of CEC2022, the results show that IA-DTPSO achieves the minimum mean rank and mean FT index in both dimensions. According to the WRST results, IA-DTPSO outperforms other algorithms with a larger number of optimization functions in pairwise comparisons. Therefore, this study utilizes IA-DTPSO to optimize the TDGM (1,1,*r*,*ξ*,*Csz*) model and applies it to simulate and predict TUWRs in China. At the same time, numerical experiments are conducted to compare IA-DTPSO with seven other algorithms and three existing models, and the results show that TDGM (1,1,*r*,*ξ*,*Csz*) optimized by IA-DTPSO obtains the smallest error among the four error evaluation indicators compared in the two groups, verifying the superiority of the proposed method. Finally, the total urban water resources in China for the next five years are predicted, and results show that by 2028, the TUWRs in China will reach 3368.846 hundred million cubic meters. In summary, the proposed IA-DTPSO achieves good results in both numerical experiments and simulation examples.

However, IA-DTPSO still has some limitations. Its performance on two functions did not surpass PSO in solving the 10-dimensional CEC2022. In addition, the accuracy obtained by IA-DTPSO in solving F3, F4, and F11 on 20-dimensional CEC2022 is not high. In the future, further improvement of IA-DTPSO’s performance on these functions can be considered. In future work, IA-DTPSO can be applied to more existing models and compared with the optimized model in this study. IA-DTPSO may not be the most perfect optimizer, but it can definitely demonstrate its applicability in a wide range of fields. It can be attempted to use IA-DTPSO to solve other complex optimization problems, such as engineering optimization [72], feature selection [73], and path planning [74].

## Figures and Tables

**Table 1:** Parameter settings of IA-DTPSO and other different types of MAs.

Algorithms	Proposed Year	Parameter	Value
PSO	1995	*ω*, *c*_1_, *c*_2_	0.8, 2, 2
RUN	2021	*a*, *b*	20, 12
NGO	2021	-	-
NOA	2023	*P_rp_*, *P_a_*_2_, *N*, δ	0.2, 0.4, 25, 0.05
GKSO	2023	*m*	1.5
IVYA	2024	-	-
EAPSO	2023	-	-
G-QPSO	2010	*ω*_1_, *ω*_2_, *c*_1_, *c*_2_	0.6, 0.8, 2, 2
HJSPSO	2023	*c*_min_, *c*_max_, *ω*_min_, *ω*_max_, *β*, *γ*, *c*_0_	0.5, 2.5, 0.4, 0.9, 0.1, 0.1, 0.5
PSO-sono	2022	*ω*_min_, *ω*_max_, *iw*, *r*	0.6, 0.8, [0.4, 0.9], 0.5
SAO-MPSO	2024	*m*, *fads*, *Jump*	1.5, 2, [0, 1]
IA-DTPSO	2025	*θ*, *a*, *ω*, *c*_1_, *c*_2_	[−1, 1], [0, 2], 0.8, 2, 2

**Table 2:** PC configuration.

Settings	Specifications
OS	Windows 11 Version 23H2 22631.4317
CPU	11th Gen Intel (R) Core (TM) i7-11700 @ 2.50 GHz
RAM	8 GB
Language (version)	Matlab (R2024a)

**Table 3:** Statistical results of IA-DTPSO and other MAs on 10-dimensional CEC2022.

F	Index	Algorithms											
		PSO	RUN	NGO	NOA	GKSO	IVYA	EAPSO	G-QPSO	HJSPSO	PSO-Sono	SAO-MPSO	IA-DTPSO
F1	Best	3.001 × 10^2^	TO	TO	2.628 × 10^3^	TO	TO	TO	1.891 × 10^3^	TO	3.875 × 10^3^	TO	TO
	Worst	3.009 × 10^2^	TO	TO	1.997 × 10^4^	TO	3.075 × 10^2^	TO	3.548 × 10^3^	TO	2.374 × 10^4^	TO	TO
	Mean	3.004 × 10^2^	TO	TO	8.435 × 10^3^	TO	3.011 × 10^2^	TO	2.679 × 10^3^	TO	1.147 × 10^4^	TO	TO
	WRST	8.007 × 10^−9^/-	8.007 × 10^−9^/-	7.992 × 10^−9^/-	8.007 × 10^−9^/-	6.054 × 10^−9^/-	8.007 × 10^−9^/-	3.338 × 10^−4^/-	8.007 × 10^−9^/-	8.007 × 10^−9^/-	8.007 × 10^−9^/-	1.427 × 10^−6^/-	-
	FT	8.800	7.100	5.300	11.300	3.675	8.100	2.275	10.000	5.700	11.700	2.675	1.375
	Rank	8	7	5	11	4	9	3	10	6	12	2	1
F2	Best	TO	TO	TO	4.562 × 10^2^	TO	TO	TO	5.000 × 10^2^	TO	4.576 × 10^2^	TO	TO
	Worst	4.073 × 10^2^	4.089 × 10^2^	4.071 × 10^2^	6.144 × 10^2^	4.089 × 10^2^	4.742 × 10^2^	4.089 × 10^2^	6.265 × 10^2^	4.041 × 10^2^	6.218 × 10^2^	4.089 × 10^2^	4.001 × 10^2^
	Mean	4.025 × 10^2^	4.036 × 10^2^	4.024 × 10^2^	5.194 × 10^2^	4.055 × 10^2^	4.101 × 10^2^	4.050 × 10^2^	5.678 × 10^2^	4.007 × 10^2^	5.004 × 10^2^	4.049 × 10^2^	TO
	WRST	4.388 × 10^−2^/-	6.949 × 10^−1^/=	5.310 × 10^−2^/=	△/-	1.604 × 10^−4^/-	6.220 × 10^−4^/-	1.135 × 10^−2^/-	△/-	1.264 × 10^−1^/=	△/-	5.842 × 10^−7^/-	-
	FT	5.300	4.400	4.750	10.900	5.750	6.350	5.475	11.800	3.850	10.250	6.125	3.050
	Rank	4	5	3	11	8	9	7	12	2	10	6	1
F3	Best	6.001 × 10^2^	6.002 × 10^2^	TO	6.111 × 10^2^	TO	TO	TO	6.246 × 10^2^	TO	6.184 × 10^2^	TO	TO
	Worst	6.103 × 10^2^	6.209 × 10^2^	TO	6.414 × 10^2^	6.100 × 10^2^	6.005 × 10^2^	TO	6.437 × 10^2^	TO	6.387 × 10^2^	6.004 × 10^2^	6.002 × 10^2^
	Mean	6.034 × 10^2^	6.097 × 10^2^	TO	6.278 × 10^2^	6.018 × 10^2^	TO	TO	6.365 × 10^2^	TO	6.260 × 10^2^	TO	TO
	WRST	1.065 × 10^−7^/-	△/-	2.946 × 10^−8^/+	△/-	2.062 × 10^−6^/-	4.355 × 10^−7^/-	2.439 × 10^−8^/+	△/-	6.810 × 10^−7^/+	△/-	6.092 × 10^−7^/-	-
	FT	8.000	8.700	3.200	10.800	7.250	1.475	2.825	11.800	4.625	10.350	3.075	5.900
	Rank	8	9	2	11	7	6	1	12	3	10	5	4
F4	Best	8.080 × 10^2^	8.109 × 10^2^	8.030 × 10^2^	8.369 × 10^2^	8.090 × 10^2^	8.090 × 10^2^	8.040 × 10^2^	8.304 × 10^2^	8.030 × 10^2^	8.397 × 10^2^	8.060 × 10^2^	8.025 × 10^2^
	Worst	8.448 × 10^2^	8.338 × 10^2^	8.107 × 10^2^	8.640 × 10^2^	8.328 × 10^2^	8.348 × 10^2^	8.318 × 10^2^	8.432 × 10^2^	8.090 × 10^2^	8.691 × 10^2^	8.259 × 10^2^	8.149 × 10^2^
	Mean	8.194 × 10^2^	8.205 × 10^2^	8.069 × 10^2^	8.512 × 10^2^	8.176 × 10^2^	8.186 × 10^2^	8.174 × 10^2^	8.374 × 10^2^	8.064 × 10^2^	8.540 × 10^2^	8.133 × 10^2^	8.083 × 10^2^
	WRST	3.293 × 10^−5^/-	1.431 × 10^−7^/-	4.388 × 10^−2^/+	△/-	1.198 × 10^−6^/-	1.200 × 10^−6^/-	1.103 × 10^−5^/-	△/-	7.114 × 10^−3^/+	△/-	5.111 × 10^−3^/-	-
	FT	6.700	7.400	2.100	11.350	6.250	6.900	6.350	10.000	1.950	11.500	4.550	2.950
	Rank	8	9	2	11	6	7	5	10	1	12	4	3
F5	Best	TO	9.023 × 10^2^	TO	9.975 × 10^2^	TO	1.007 × 10^3^	TO	1.079 × 10^3^	TO	1.001 × 10^3^	TO	TO
	Worst	9.001 × 10^2^	1.021 × 10^3^	9.001 × 10^2^	1.381 × 10^3^	9.017 × 10^2^	1.710 × 10^3^	9.005 × 10^2^	1.182 × 10^3^	9.005 × 10^2^	1.366 × 10^3^	9.005 × 10^2^	9.006 × 10^2^
	Mean	TO	9.714 × 10^2^	TO	1.199 × 10^3^	9.003 × 10^2^	1.243 × 10^3^	TO	1.110 × 10^3^	TO	1.161 × 10^3^	9.001 × 10^2^	TO
	WRST	2.745 × 10^−4^/+	△/-	9.996 × 10^−7^/+	△/-	4.088 × 10^−1^/=	△/-	6.326 × 10^−6^/-	△/-	4.703 × 10^−3^/+	△/-	2.033 × 10^−2^/-	-
	FT	5.650	8.000	2.875	11.000	4.900	10.900	1.600	9.750	4.550	10.350	3.375	5.050
	Rank	2	8	1	11	7	12	5	9	3	10	6	4
F6	Best	1.866 × 10^3^	1.903 × 10^3^	1.828 × 10^3^	8.824 × 10^5^	1.826 × 10^3^	1.857 × 10^3^	1.942 × 10^3^	3.479 × 10^5^	1.843 × 10^3^	4.901 × 10^5^	1.902 × 10^3^	TO
	Worst	6.960 × 10^3^	4.950 × 10^3^	1.943 × 10^3^	3.144 × 10^7^	5.528 × 10^3^	8.090 × 10^3^	7.181 × 10^3^	4.242 × 10^6^	3.069 × 10^3^	2.214 × 10^7^	7.657 × 10^3^	1.803 × 10^3^
	Mean	3.069 × 10^3^	3.096 × 10^3^	1.882 × 10^3^	8.279 × 10^6^	2.236 × 10^3^	4.031 × 10^3^	4.335 × 10^3^	2.007 × 10^6^	2.109 × 10^3^	6.362 × 10^6^	4.436 × 10^3^	1.801 × 10^3^
	WRST	△/-	△/-	△/-	△/-	△/-	△/-	△/-	△/-	△/-	△/-	△/-	-
	FT	5.700	6.450	2.750	11.350	3.950	6.850	7.150	10.500	4.150	11.150	7.000	1.000
	Rank	5	6	2	12	4	7	8	10	3	11	9	1
F7	Best	2.002 × 10^3^	2.017 × 10^3^	2.001 × 10^3^	2.046 × 10^3^	2.002 × 10^3^	2.001 × 10^3^	TO	2.070 × 10^3^	2.001 × 10^3^	2.051 × 10^3^	2.001 × 10^3^	2.001 × 10^3^
	Worst	2.045 × 10^3^	2.058 × 10^3^	2.011 × 10^3^	2.096 × 10^3^	2.026 × 10^3^	2.084 × 10^3^	2.054 × 10^3^	2.105 × 10^3^	2.025 × 10^3^	2.121 × 10^3^	2.025 × 10^3^	2.022 × 10^3^
	Mean	2.028 × 10^3^	2.036 × 10^3^	2.004 × 10^3^	2.072 × 10^3^	2.020 × 10^3^	2.021 × 10^3^	2.017 × 10^3^	2.088 × 10^3^	2.010 × 10^3^	2.085 × 10^3^	2.012 × 10^3^	2.009 × 10^3^
	WRST	2.062 × 10^−6^/-	1.431 × 10^−7^/-	1.782 × 10^−3^/+	△/-	1.610 × 10^−4^/-	1.143 × 10^−2^/-	1.404 × 10^−1^/=	△/-	8.392 × 10^−1^/=	△/-	8.392 × 10^−1^/=	-
	FT	7.500	8.000	2.050	10.300	5.700	5.700	4.650	11.400	4.100	11.200	3.800	3.600
	Rank	8	9	1	10	6	7	5	12	3	11	4	2
F8	Best	2.202 × 10^3^	2.204 × 10^3^	2.208 × 10^3^	2.228 × 10^3^	TO	2.201 × 10^3^	TO	2.224 × 10^3^	2.210 × 10^3^	2.221 × 10^3^	TO	2.207 × 10^3^
	Worst	2.228 × 10^3^	2.226 × 10^3^	2.223 × 10^3^	2.245 × 10^3^	2.221 × 10^3^	2.224 × 10^3^	2.222 × 10^3^	2.235 × 10^3^	2.227 × 10^3^	2.312 × 10^3^	2.221 × 10^3^	2.215 × 10^3^
	Mean	2.223 × 10^3^	2.222 × 10^3^	2.218 × 10^3^	2.237 × 10^3^	2.216 × 10^3^	2.219 × 10^3^	2.220 × 10^3^	2.232 × 10^3^	2.223 × 10^3^	2.246 × 10^3^	2.216 × 10^3^	2.210 × 10^3^
	WRST	1.201 × 10^−6^/-	1.201 × 10^−6^/-	3.705 × 10^−5^/-	△/-	7.114 × 10^−3^/-	1.807 × 10^−5^/-	1.201 × 10^−6^/-	△/-	4.539 × 10^−7^/-	△/-	7.114 × 10^−3^/-	-
	FT	7.250	7.100	4.800	11.150	3.400	5.200	4.500	10.100	7.550	11.500	3.450	2.000
	Rank	8	7	4	11	3	5	6	10	8	12	2	1
F9	Best	2.486 × 10^3^	2.529 × 10^3^	2.529 × 10^3^	2.570 × 10^3^	2.529 × 10^3^	2.529 × 10^3^	2.529 × 10^3^	2.643 × 10^3^	2.529 × 10^3^	2.586 × 10^3^	2.529 × 10^3^	2.486 × 10^3^
	Worst	2.486 × 10^3^	2.529 × 10^3^	2.529 × 10^3^	2.710 × 10^3^	2.529 × 10^3^	2.676 × 10^3^	2.529 × 10^3^	2.669 × 10^3^	2.529 × 10^3^	2.683 × 10^3^	2.529 × 10^3^	2.490 × 10^3^
	Mean	2.486 × 10^3^	2.529 × 10^3^	2.529 × 10^3^	2.620 × 10^3^	2.529 × 10^3^	2.537 × 10^3^	2.529 × 10^3^	2.659 × 10^3^	2.529 × 10^3^	2.640 × 10^3^	2.529 × 10^3^	2.488 × 10^3^
	WRST	△/+	△/-	1.127 × 10^−8^/-	△/-	6.777 × 10^−8^/-	5.366 × 10^−8^/-	8.007 × 10^−9^/-	△/-	6.644 × 10^−8^/-	△/-	1.945 × 10^−8^/-	-
	FT	1.000	8.850	4.250	10.400	7.525	6.375	4.175	11.550	6.550	10.900	4.425	2.000
	Rank	1	8	4	10	7	9	3	12	6	11	5	2
F10	Best	TO	TO	TO	2.503 × 10^3^	TO	TO	TO	2.508 × 10^3^	TO	2.502 × 10^3^	TO	TO
	Worst	2.633 × 10^3^	2.619 × 10^3^	TO	2.676 × 10^3^	TO	2.638 × 10^3^	2.618 × 10^3^	2.651 × 10^3^	TO	2.684 × 10^3^	TO	TO
	Mean	2.555 × 10^3^	2.534 × 10^3^	TO	2.533 × 10^3^	TO	2.541 × 10^3^	2.512 × 10^3^	2.539 × 10^3^	TO	2.547 × 10^3^	TO	TO
	WRST	△/-	6.674 × 10^−6^/-	8.604 × 10^−1^/=	△/-	6.868 × 10^−4^/-	4.540 × 10^−6^/-	1.105 × 10^−5^/-	△/-	3.048 × 10^−4^/-	△/-	6.750 × 10^−1^/=	-
	FT	6.750	8.100	4.000	9.950	3.550	7.950	6.550	10.400	5.650	9.600	3.650	1.850
	Rank	12	8	5	7	2	10	6	9	3	11	4	1
F11	Best	2.601 × 10^3^	TO	TO	2.762 × 10^3^	TO	TO	TO	2.822 × 10^3^	TO	2.769 × 10^3^	TO	TO
	Worst	3.001 × 10^3^	3.184 × 10^3^	TO	2.871 × 10^3^	3.000 × 10^3^	3.000 × 10^3^	3.000 × 10^3^	2.899 × 10^3^	TO	3.429 × 10^3^	3.184 × 10^3^	TO
	Mean	2.672 × 10^3^	2.659 × 10^3^	TO	2.815 × 10^3^	2.640 × 10^3^	2.768 × 10^3^	2.673 × 10^3^	2.865 × 10^3^	TO	2.835 × 10^3^	2.964 × 10^3^	TO
	WRST	3.473 × 10^−8^/-	3.473 × 10^−8^/-	2.512 × 10^−1^/=	3.473 × 10^−8^/-	1.889 × 10^−4^/-	6.682 × 10^−5^/-	5.164 × 10^−2^/=	3.473 × 10^−8^/-	1.512 × 10^−5^/-	3.473 × 10^−8^/-	8.221 × 10^−6^/-	-
	FT	7.750	6.750	2.400	8.900	4.675	7.100	4.075	10.050	5.200	8.900	9.900	2.300
	Rank	6	5	2	9	4	8	7	11	3	10	12	1
F12	Best	2.801 × 10^3^	2.862 × 10^3^	2.859 × 10^3^	2.872 × 10^3^	2.863 × 10^3^	2.864 × 10^3^	2.863 × 10^3^	2.931 × 10^3^	2.865 × 10^3^	2.875 × 10^3^	2.862 × 10^3^	2.846 × 10^3^
	Worst	2.926 × 10^3^	2.867 × 10^3^	2.864 × 10^3^	2.967 × 10^3^	2.868 × 10^3^	2.920 × 10^3^	2.866 × 10^3^	2.966 × 10^3^	2.871 × 10^3^	3.000 × 10^3^	2.866 × 10^3^	2.849 × 10^3^
	Mean	2.857 × 10^3^	2.864 × 10^3^	2.862 × 10^3^	2.891 × 10^3^	2.865 × 10^3^	2.871 × 10^3^	2.864 × 10^3^	2.948 × 10^3^	2.866 × 10^3^	2.898 × 10^3^	2.864 × 10^3^	2.847 × 10^3^
	WRST	1.803 × 10^−6^/-	△/-	△/-	△/-	6.786 × 10^−8^/-	6.757 × 10^−8^/-	6.786 × 10^−8^/-	△/-	△/-	△/-	6.786 × 10^−8^/-	-
	FT	2.550	5.300	3.300	10.450	5.900	8.600	5.150	11.850	7.600	10.400	5.800	1.100
	Rank	2	6	3	10	7	9	5	12	8	11	4	1
Mean Rank		6.000	7.250	2.833	10.333	5.083	8.167	5.083	10.750	4.083	10.917	5.250	1.833
Final Ranking		7	8	2	10	4	9	4	11	3	12	6	1
Mean FT		6.079	7.179	3.481	10.654	5.210	6.792	4.565	10.767	5.123	10.650	4.819	2.681
Final FT		7	9	2	11	6	8	3	12	5	10	4	1
+/=/−		2/0/10	0/1/11	4/3/5	0/0/12	0/1/11	0/0/12	1/2/9	0/0/12	3/2/7	0/0/12	0/2/10	-/-/-

△: 6.796 × 10^−8^ is replaced by △.

**Table 4:** Errors of IA-DTPSO and other MAs on 10-dimensional CEC2022.

F	Index	Algorithms											
		PSO	RUN	NGO	NOA	GKSO	IVYA	EAPSO	G-QPSO	HJSPSO	PSO-Sono	SAO-MPSO	IA-DTPSO
F1	Std	2.461 × 10^−1^	1.625 × 10^−5^	5.384 × 10^−10^	4.211 × 10^3^	1.258 × 10^−13^	2.249	4.124 × 10^−14^	4.362 × 10^2^	3.157 × 10^−9^	5.113 × 10^3^	2.916 × 10^−14^	0.000
	RMSE	2.994 × 10^2^	2.990 × 10^2^	2.990 × 10^2^	9.379 × 10^3^	2.990 × 10^2^	3.001 × 10^2^	2.990 × 10^2^	2.711 × 10^3^	2.990 × 10^2^	1.251 × 104	2.990 × 10^2^	2.990 × 10^2^
	*δ*	2.991 × 10^2^	2.990 × 10^2^	2.990 × 10^2^	2.627 × 10^3^	2.990 × 10^2^	2.990 × 10^2^	2.990 × 10^2^	1.890 × 10^3^	2.990 × 10^2^	3.874 × 10^3^	2.990 × 10^2^	2.990 × 10^2^
F2	Std	2.265	4.481	2.538	3.991 × 10^1^	3.834	2.175 × 10^1^	3.660	3.138 × 10^1^	1.458	3.467 × 10^1^	4.042	3.083 × 10^−2^
	RMSE	4.015 × 10^2^	4.026 × 10^2^	3.990 × 10^2^	5.198 × 10^2^	4.045 × 10^2^	4.096 × 10^2^	4.040 × 10^2^	5.676 × 10^2^	3.997 × 10^2^	5.005 × 10^2^	4.039 × 10^2^	4.014 × 10^2^
	*δ*	3.990 × 10^2^	3.990 × 10^2^	3.990 × 10^2^	4.552 × 10^2^	3.990 × 10^2^	3.990 × 10^2^	3.990 × 10^2^	4.990 × 10^2^	3.990 × 10^2^	4.566 × 10^2^	3.990 × 10^2^	3.990 × 10^2^
F3	Std	2.602	7.359	1.744 × 10^−6^	7.342	2.593	1.228 × 10^−1^	6.901 × 10^−14^	3.930	4.006 × 10^−3^	4.209	9.164 × 10^−2^	4.160 × 10^−2^
	RMSE	6.024 × 10^2^	6.087 × 10^2^	5.990 × 10^2^	6.268 × 10^2^	6.008 × 10^2^	5.990 × 10^2^	5.990 × 10^2^	6.355 × 10^2^	5.990 × 10^2^	6.250 × 10^2^	5.990 × 10^2^	5.990 × 10^2^
	*δ*	5.991 × 10^2^	5.992 × 10^2^	5.990 × 10^2^	6.101 × 10^2^	5.990 × 10^2^	5.990 × 10^2^	5.990 × 10^2^	6.236 × 10^2^	5.990 × 10^2^	6.174 × 10^2^	5.990 × 10^2^	5.990 × 10^2^
F4	Std	9.612	5.622	2.012	7.638	6.515	6.833	6.587	3.618	1.618	8.390	6.020	2.867
	RMSE	8.185 × 10^2^	8.196 × 10^2^	8.059 × 10^2^	8.503 × 10^2^	8.166 × 10^2^	8.176 × 10^2^	8.165 × 10^2^	8.364 × 10^2^	8.054 × 10^2^	8.531 × 10^2^	8.124 × 10^2^	8.073 × 10^2^
	*δ*	8.070 × 10^2^	8.099 × 10^2^	8.020 × 10^2^	8.359 × 10^2^	8.080 × 10^2^	8.080 × 10^2^	8.030 × 10^2^	8.294 × 10^2^	8.020 × 10^2^	8.387 × 10^2^	8.050 × 10^2^	8.015 × 10^2^
F5	Std	3.486 × 10^−2^	3.548 × 10^1^	2.002 × 10^−2^	1.137 × 10^2^	4.573 × 10^−1^	1.707 × 10^2^	1.543 × 10^−1^	2.964 × 10^1^	1.139 × 10^−1^	8.556 × 10^1^	1.385 × 10^−1^	1.334 × 10^−1^
	RMSE	8.990 × 10^2^	9.710 × 10^2^	8.990 × 10^2^	1.203 × 10^3^	8.993 × 10^2^	1.254 × 10^3^	8.990 × 10^2^	1.109 × 10^3^	8.990 × 10^2^	1.163 × 10^3^	8.991 × 10^2^	8.990 × 10^2^
	*δ*	8.990 × 10^2^	9.013 × 10^2^	8.990 × 10^2^	9.965 × 10^2^	8.990 × 10^2^	1.006 × 10^3^	8.990 × 10^2^	1.078 × 10^3^	8.990 × 10^2^	9.998 × 10^2^	8.990 × 10^2^	8.990 × 10^2^
F6	Std	1.600 × 10^3^	1.128 × 10^3^	3.221 × 10^1^	9.251 × 10^6^	8.287 × 10^2^	2.139 × 10^3^	2.003 × 10^3^	1.298 × 10^6^	3.062 × 10^2^	5.700 × 10^6^	2.261 × 10^3^	9.067 × 10^−1^
	RMSE	3.442 × 10^3^	3.284 × 10^3^	1.881 × 10^3^	1.224 × 10^7^	2.376 × 10^3^	4.537 × 10^3^	4.753 × 10^3^	2.372 × 10^6^	2.129 × 10^3^	8.446 × 10^6^	4.952 × 10^3^	TO
	*δ*	1.865 × 10^3^	1.902 × 10^3^	1.827 × 10^3^	8.824 × 10^5^	1.825 × 10^3^	1.856 × 10^3^	1.941 × 10^3^	3.479 × 10^5^	1.842 × 10^3^	4.901 × 10^5^	1.901 × 10^3^	1.799 × 10^3^
F7	Std	9.656	1.171 × 10^1^	3.449	1.378 × 10^1^	6.571	1.823 × 10^1^	1.383 × 10^1^	9.648	9.078	1.718 × 10^1^	9.943	5.864
	RMSE	2.027 × 10^3^	2.035 × 10^3^	2.003 × 10^3^	2.071 × 10^3^	2.019 × 10^3^	2.020 × 10^3^	2.016 × 10^3^	2.087 × 10^3^	2.009 × 10^3^	2.084 × 10^3^	2.011 × 10^3^	2.008 × 10^3^
	*δ*	2.001 × 10^3^	2.016 × 10^3^	TO	2.045 × 10^3^	2.001 × 10^3^	TO	1.999 × 10^3^	2.069 × 10^3^	TO	2.050 × 10^3^	TO	TO
F8	Std	5.340	4.507	5.146	4.450	8.882	5.876	4.653	2.753	4.466	1.754 × 10^1^	8.845	2.329
	RMSE	2.222 × 10^3^	2.221 × 10^3^	2.217 × 10^3^	2.236 × 10^3^	2.215 × 10^3^	2.218 × 10^3^	2.219 × 10^3^	2.231 × 10^3^	2.222 × 10^3^	2.246 × 10^3^	2.215 × 10^3^	2.209 × 10^3^
	*δ*	2.201 × 10^3^	2.203 × 10^3^	2.207 × 10^3^	2.227 × 10^3^	2.199 × 10^3^	TO	2.199 × 10^3^	2.223 × 10^3^	2.209 × 10^3^	2.220 × 10^3^	2.199 × 10^3^	2.206 × 10^3^
F9	Std	1.141 × 10^−3^	3.325 × 10^−5^	1.043 × 10^−13^	3.961 × 10^1^	4.092 × 10^−9^	3.286 × 10^1^	0.000	8.315	5.291 × 10^−12^	2.874 × 10^1^	1.807 × 10^−13^	1.108
	RMSE	2.485 × 10^3^	2.528 × 10^3^	2.528 × 10^3^	2.619 × 10^3^	2.528 × 10^3^	2.536 × 10^3^	2.528 × 10^3^	2.659 × 10^3^	2.528 × 10^3^	2.639 × 10^3^	2.528 × 10^3^	2.487 × 10^3^
	*δ*	2.485 × 10^3^	2.528 × 10^3^	2.528 × 10^3^	2.569 × 10^3^	2.528 × 10^3^	2.528 × 10^3^	2.528 × 10^3^	2.642 × 10^3^	2.528 × 10^3^	2.585 × 10^3^	2.528 × 10^3^	2.485 × 10^3^
F10	Std	6.277 × 10^1^	5.267 × 10^1^	8.037 × 10^−2^	5.130 × 10^1^	5.454 × 10^−2^	5.760 × 10^1^	3.488 × 10^1^	4.883 × 10^1^	7.003 × 10^−2^	7.598 × 10^1^	7.548 × 10^−2^	5.131 × 10^−2^
	RMSE	2.555 × 10^3^	2.534 × 10^3^	2.499 × 10^3^	2.532 × 10^3^	2.499 × 10^3^	2.541 × 10^3^	2.511 × 10^3^	2.538 × 10^3^	2.499 × 10^3^	2.547 × 10^3^	2.499 × 10^3^	2.499 × 10^3^
	*δ*	2.499 × 10^3^	2.499 × 10^3^	2.499 × 10^3^	2.502 × 10^3^	2.499 × 10^3^	2.499 × 10^3^	2.499 × 10^3^	2.507 × 10^3^	2.499 × 10^3^	2.501 × 10^3^	2.499 × 10^3^	2.499 × 10^3^
F11	Std	1.455 × 10^2^	1.378 × 10^2^	6.211 × 10^−10^	3.421 × 10^1^	1.231 × 10^2^	1.808 × 10^2^	1.352 × 10^2^	2.235 × 10^1^	2.612 × 10^−9^	1.603 × 10^2^	1.852 × 10^2^	3.460 × 10^−13^
	RMSE	2.674 × 10^3^	2.662 × 10^3^	2.599 × 10^3^	2.815 × 10^3^	2.642 × 10^3^	2.772 × 10^3^	2.675 × 10^3^	2.864 × 10^3^	2.599 × 10^3^	2.839 × 10^3^	2.969 × 10^3^	2.599 × 10^3^
	*δ*	TO	2.599 × 10^3^	2.599 × 10^3^	2.761 × 10^3^	2.599 × 10^3^	2.599 × 10^3^	2.599 × 10^3^	2.821 × 10^3^	2.599 × 10^3^	2.768 × 10^3^	2.599 × 10^3^	2.599 × 10^3^
F12	Std	2.080 × 10^1^	1.145	1.688	2.276 × 10^1^	1.362	1.232 × 10^1^	1.064	9.563	1.749	3.320 × 10^1^	9.740 × 10^−1^	7.480 × 10^−1^
	RMSE	2.856 × 10^3^	2.863 × 10^3^	2.861 × 10^3^	2.890 × 10^3^	2.864 × 10^3^	2.870 × 10^3^	2.863 × 10^3^	2.947 × 10^3^	2.865 × 10^3^	2.897 × 10^3^	2.863 × 10^3^	2.846 × 10^3^
	*δ*	2.800 × 10^3^	2.861 × 10^3^	2.858 × 10^3^	2.871 × 10^3^	2.862 × 10^3^	2.863 × 10^3^	2.862 × 10^3^	2.930 × 10^3^	2.864 × 10^3^	2.874 × 10^3^	2.861 × 10^3^	2.845 × 10^3^

**Table 5:** Statistical results of IA-DTPSO and other MAs on 20-dimensional CEC2022.

F	Index	Algorithms											
		PSO	RUN	NGO	NOA	GKSO	IVYA	EAPSO	G-QPSO	HJSPSO	PSO-Sono	SAO-MPSO	IA-DTPSO
F1	Best	3.153 × 10^2^	TO	2.016 × 10^3^	2.665 × 10^4^	TO	5.133 × 10^3^	TO	1.452 × 10^4^	6.166 × 10^2^	2.779 × 10^4^	TO	TO
	Worst	3.434 × 10^2^	TO	6.215 × 10^3^	6.186 × 10^4^	TO	1.751 × 10^4^	TO	2.237 × 10^4^	2.105 × 10^3^	9.624 × 10^4^	TO	TO
	Mean	3.247 × 10^2^	TO	4.014 × 10^3^	3.917 × 10^4^	TO	9.801 × 10^3^	TO	1.873 × 10^4^	1.314 × 10^3^	5.183 × 10^4^	TO	TO
	WRST	△/-	△/-	△/-	△/-	△/-	△/-	5.075 × 10^−1^/=	△/-	△/-	△/-	6.653 × 10^−8^/+	-
	FT	6.000	4.000	8.000	11.200	5.000	9.000	2.750	10.000	7.000	11.800	1.000	2.250
	Rank	6	4	8	11	5	9	3	10	7	12	1	2
F2	Best	4.154 × 10^2^	TO	4.002 × 10^2^	8.056 × 10^2^	TO	4.289 × 10^2^	TO	9.092 × 10^2^	4.449 × 10^2^	7.176 × 10^2^	4.026 × 10^2^	4.105 × 10^2^
	Worst	4.753 × 10^2^	4.491 × 10^2^	4.747 × 10^2^	1.499 × 10^3^	4.685 × 10^2^	4.723 × 10^2^	4.491 × 10^2^	1.118 × 10^3^	4.755 × 10^2^	1.157 × 10^3^	4.686 × 10^2^	4.316 × 10^2^
	Mean	4.429 × 10^2^	4.417 × 10^2^	4.536 × 10^2^	1.045 × 10^3^	4.402 × 10^2^	4.522 × 10^2^	4.388 × 10^2^	1.025 × 10^3^	4.546 × 10^2^	9.211 × 10^2^	4.462 × 10^2^	4.238 × 10^2^
	WRST	8.182 × 10^−1^/=	1.610 × 10^−4^/-	1.201 × 10^−6^/-	△/-	1.481 × 10^−3^/-	7.898 × 10^−8^/-	1.217 × 10^−3^/-	△/-	△/-	△/-	1.587 × 10^−5^/-	-
	FT	4.900	4.650	7.050	11.300	4.500	6.650	3.050	11.350	7.600	10.350	4.300	2.300
	Rank	5	4	8	12	3	7	2	11	9	10	6	1
F3	Best	6.154 × 10^2^	6.164 × 10^2^	TO	6.294 × 10^2^	6.087 × 10^2^	TO	TO	6.518 × 10^2^	TO	6.291 × 10^2^	TO	6.026 × 10^2^
	Worst	6.459 × 10^2^	6.431 × 10^2^	6.002 × 10^2^	6.817 × 10^2^	6.324 × 10^2^	6.223 × 10^2^	TO	6.686 × 10^2^	6.033 × 10^2^	6.675 × 10^2^	6.030 × 10^2^	6.181 × 10^2^
	Mean	6.337 × 10^2^	6.277 × 10^2^	TO	6.585 × 10^2^	6.184 × 10^2^	6.020 × 10^2^	TO	6.630 × 10^2^	6.006 × 10^2^	6.511 × 10^2^	6.004 × 10^2^	6.063 × 10^2^
	WRST	1.235 × 10^−7^/-	2.218 × 10^−7^/-	△/+	△/-	2.690 × 10^−6^/-	1.251 × 10^−5^/+	△/+	△/-	7.898 × 10^−8^/+	△/-	7.898 × 10^−8^/+	-
	FT	8.700	8.000	3.000	10.950	7.200	2.500	1.550	11.550	4.350	10.400	3.800	6.000
	Rank	9	8	2	11	7	5	1	12	4	10	3	6
F4	Best	8.399 × 10^2^	8.438 × 10^2^	8.247 × 10^2^	9.495 × 10^2^	8.328 × 10^2^	8.428 × 10^2^	8.199 × 10^2^	9.244 × 10^2^	8.195 × 10^2^	9.414 × 10^2^	8.139 × 10^2^	8.319 × 10^2^
	Worst	8.897 × 10^2^	8.955 × 10^2^	8.515 × 10^2^	9.987 × 10^2^	9.025 × 10^2^	8.866 × 10^2^	8.955 × 10^2^	9.565 × 10^2^	8.468 × 10^2^	1.008 × 10^3^	8.557 × 10^2^	8.762 × 10^2^
	Mean	8.642 × 10^2^	8.739 × 10^2^	8.396 × 10^2^	9.745 × 10^2^	8.643 × 10^2^	8.655 × 10^2^	8.414 × 10^2^	9.444 × 10^2^	8.315 × 10^2^	9.737 × 10^2^	8.318 × 10^2^	8.531 × 10^2^
	WRST	2.074 × 10^−2^/-	4.680 × 10^−5^/-	3.382 × 10^−4^/+	△/-	6.557 × 10^−3^/-	4.320 × 10^−3^/-	8.355 × 10^−3^/+	△/-	7.948 × 10^−7^/+	△/-	9.278 × 10^−5^/+	-
	FT	6.650	7.950	3.400	11.300	6.800	6.950	3.550	10.150	2.000	11.550	2.500	5.200
	Rank	6	9	3	12	7	8	4	10	1	11	2	5
F5	Best	9.019 × 10^2^	1.341 × 10^3^	9.081 × 10^2^	2.247 × 10^3^	9.091 × 10^2^	1.940 × 10^3^	TO	2.579 × 10^3^	9.001 × 10^2^	2.379 × 10^3^	9.002 × 10^2^	9.006 × 10^2^
	Worst	2.213 × 10^3^	2.316 × 10^3^	1.514 × 10^3^	4.371 × 10^3^	2.304 × 10^3^	2.498 × 10^3^	9.258 × 10^2^	3.076 × 10^3^	9.551 × 10^2^	5.071 × 10^3^	1.590 × 10^3^	9.079 × 10^2^
	Mean	1.511 × 10^3^	1.732 × 10^3^	1.186 × 10^3^	3.080 × 10^3^	1.306 × 10^3^	2.295 × 10^3^	9.015 × 10^2^	2.780 × 10^3^	9.061 × 10^2^	3.171 × 10^3^	1.017 × 10^3^	9.042 × 10^2^
	WRST	4.680 × 10^−5^/-	△/-	△/-	△/-	△/-	△/-	1.306 × 10^−6^/+	△/-	1.404 × 10^−1^/=	△/-	4.903 × 10^−1^/=	-
	FT	6.700	7.400	5.300	11.100	5.950	8.950	1.250	10.550	3.200	11.250	3.750	2.600
	Rank	7	8	5	11	6	9	1	10	3	12	4	2
F6	Best	5.703 × 10^3^	1.923 × 10^3^	2.288 × 10^3^	7.281 × 10^7^	1.864 × 10^3^	1.926 × 10^3^	1.930 × 10^3^	3.348 × 10^7^	1.842 × 10^3^	9.198 × 10^7^	1.947 × 10^3^	1.815 × 10^3^
	Worst	7.610 × 10^4^	4.447 × 10^3^	4.619 × 10^3^	5.995 × 10^8^	2.266 × 10^4^	5.921 × 10^3^	2.277 × 10^4^	1.987 × 10^8^	4.698 × 10^3^	3.962 × 10^8^	2.505 × 10^4^	1.919 × 10^3^
	Mean	2.908 × 10^4^	3.547 × 10^3^	3.083 × 10^3^	2.420 × 10^8^	1.002 × 10^4^	3.351 × 10^3^	8.944 × 10^3^	1.201 × 10^8^	2.769 × 10^3^	2.049 × 10^8^	9.325 × 10^3^	1.833 × 10^3^
	WRST	△/-	△/-	△/-	△/-	9.173 × 10^−8^/-	△/-	△/-	△/-	1.431 × 10^−7^/-	△/-	△/-	-
	FT	8.750	5.000	4.400	11.400	6.250	4.300	5.900	10.350	3.400	11.250	5.900	1.100
	Rank	9	5	3	12	8	4	6	10	2	11	7	1
F7	Best	2.048 × 10^3^	2.045 × 10^3^	2.045 × 10^3^	2.143 × 10^3^	2.027 × 10^3^	2.067 × 10^3^	2.021 × 10^3^	2.163 × 10^3^	2.029 × 10^3^	2.168 × 10^3^	2.021 × 10^3^	2.045 × 10^3^
	Worst	2.167 × 10^3^	2.142 × 10^3^	2.085 × 10^3^	2.318 × 10^3^	2.142 × 10^3^	2.155 × 10^3^	2.172 × 10^3^	2.195 × 10^3^	2.059 × 10^3^	2.352 × 10^3^	2.181 × 10^3^	2.072 × 10^3^
	Mean	2.099 × 10^3^	2.109 × 10^3^	2.064 × 10^3^	2.209 × 10^3^	2.070 × 10^3^	2.112 × 10^3^	2.062 × 10^3^	2.182 × 10^3^	2.045 × 10^3^	2.244 × 10^3^	2.068 × 10^3^	2.056 × 10^3^
	WRST	1.600 × 10^−5^/-	1.803 × 10^−6^/-	4.679 × 10^−2^/-	△/-	1.332 × 10^−2^/-	1.235 × 10^−7^/-	5.979 × 10^−1^/=	△/-	1.481 × 10^−3^/+	△/-	9.892 × 10^−1^/=	-
	FT	6.550	7.650	4.500	10.950	5.100	7.850	3.750	10.300	2.200	11.600	3.850	3.700
	Rank	7	8	4	11	6	9	3	10	1	12	5	2
F8	Best	2.225 × 10^3^	2.223 × 10^3^	2.223 × 10^3^	2.250 × 10^3^	2.221 × 10^3^	2.221 × 10^3^	2.221 × 10^3^	2.238 × 10^3^	2.225 × 10^3^	2.239 × 10^3^	2.221 × 10^3^	2.216 × 10^3^
	Worst	2.363 × 10^3^	2.243 × 10^3^	2.229 × 10^3^	2.424 × 10^3^	2.341 × 10^3^	2.576 × 10^3^	2.358 × 10^3^	2.263 × 10^3^	2.236 × 10^3^	2.532 × 10^3^	2.240 × 10^3^	2.231 × 10^3^
	Mean	2.243 × 10^3^	2.227 × 10^3^	2.227 × 10^3^	TO	2.232 × 10^3^	2.329 × 10^3^	2.264 × 10^3^	2.253 × 10^3^	2.230 × 10^3^	2.376 × 10^3^	2.227 × 10^3^	2.226 × 10^3^
	WRST	1.116 × 10^−3^/-	5.250 × 10^−1^/=	9.031 × 10^−1^/=	△/-	6.787 × 10^−2^/=	8.292 × 10^−5^/-	5.979 × 10^−1^/=	△/-	1.159 × 10^−4^/-	△/-	1.636 × 10^−1^/=	-
	FT	6.550	4.350	4.500	10.300	3.550	9.200	5.500	8.950	6.150	11.250	4.300	3.400
	Rank	7	3	2	10	6	11	9	8	5	12	4	1
F9	Best	2.465 × 10^3^	2.481 × 10^3^	2.481 × 10^3^	2.565 × 10^3^	2.481 × 10^3^	2.481 × 10^3^	2.481 × 10^3^	2.712 × 10^3^	2.481 × 10^3^	2.579 × 10^3^	2.481 × 10^3^	2.472 × 10^3^
	Worst	2.465 × 10^3^	2.481 × 10^3^	2.481 × 10^3^	2.775 × 10^3^	2.481 × 10^3^	2.482 × 10^3^	2.481 × 10^3^	2.916 × 10^3^	2.481 × 10^3^	2.816 × 10^3^	2.481 × 10^3^	2.481 × 10^3^
	Mean	2.465 × 10^3^	2.481 × 10^3^	2.481 × 10^3^	2.650 × 10^3^	2.481 × 10^3^	2.481 × 10^3^	2.481 × 10^3^	2.809 × 10^3^	2.481 × 10^3^	2.703 × 10^3^	2.481 × 10^3^	2.476 × 10^3^
	WRST	△/+	△/-	△/-	△/-	△/-	△/-	5.903 × 10^−8^/-	△/-	△/-	△/-	6.541 × 10^−8^/-	-
	FT	1.000	7.550	5.300	10.200	5.600	9.000	3.200	11.850	7.450	10.950	3.900	2.000
	Rank	1	8	4	10	6	9	3	12	7	11	5	2
F10	Best	TO	2.501 × 10^3^	TO	2.538 × 10^3^	TO	TO	2.501 × 10^3^	2.601 × 10^3^	2.501 × 10^3^	2.522 × 10^3^	TO	TO
	Worst	4.867 × 10^3^	2.627 × 10^3^	2.625 × 10^3^	6.769 × 10^3^	2.711 × 10^3^	5.023 × 10^3^	3.985 × 10^3^	2.674 × 10^3^	2.637 × 10^3^	7.760 × 10^3^	4.510 × 10^3^	2.501 × 10^3^
	Mean	3.639 × 10^3^	2.507 × 10^3^	2.507 × 10^3^	3.024 × 10^3^	2.511 × 10^3^	3.197 × 10^3^	2.886 × 10^3^	2.634 × 10^3^	2.508 × 10^3^	4.594 × 10^3^	3.056 × 10^3^	2.501 × 10^3^
	WRST	1.227 × 10^−3^/-	2.218 × 10^−7^/-	7.205 × 10^−2^/=	△/-	2.561 × 10^−3^/-	1.929 × 10^−2^/-	4.540 × 10^−6^/-	△/-	1.794 × 10^−4^/-	△/-	5.874 × 10^−6^/-	-
	FT	8.900	5.950	3.650	8.350	2.050	6.600	7.750	8.600	4.750	9.500	9.050	2.850
	Rank	11	3	2	8	5	10	7	6	4	12	9	1
F11	Best	2.651 × 10^3^	2.900 × 10^3^	TO	4.176 × 10^3^	2.900 × 10^3^	2.900 × 10^3^	2.900 × 10^3^	6.225 × 10^3^	TO	3.676 × 10^3^	2.900 × 10^3^	TO
	Worst	3.008 × 10^3^	3.000 × 10^3^	3.000 × 10^3^	7.276 × 10^3^	3.360 × 10^3^	3.000 × 10^3^	3.000 × 10^3^	7.128 × 10^3^	3.000 × 10^3^	5.998 × 10^3^	2.900 × 10^3^	3.038 × 10^3^
	Mean	2.950 × 10^3^	2.910 × 10^3^	2.888 × 10^3^	5.612 × 10^3^	2.963 × 10^3^	2.930 × 10^3^	2.945 × 10^3^	6.742 × 10^3^	2.885 × 10^3^	4.959 × 10^3^	2.900 × 10^3^	2.922 × 10^3^
	WRST	3.852 × 10^−2^/-	7.557 × 10^−1^/=	3.382 × 10^−4^/+	△/-	8.103 × 10^−2^/=	3.639 × 10^−3^/-	5.231 × 10^−2^/=	△/-	2.561 × 10^−3^/+	△/-	7.656 × 10^−7^/+	-
	FT	7.050	6.250	4.500	10.850	5.500	4.400	4.350	11.950	5.150	10.200	1.350	6.450
	Rank	8	4	2	11	9	6	7	12	1	10	3	5
F12	Best	2.896 × 10^3^	2.941 × 10^3^	2.935 × 10^3^	3.113 × 10^3^	2.944 × 10^3^	2.947 × 10^3^	2.934 × 10^3^	3.469 × 10^3^	2.954 × 10^3^	3.059 × 10^3^	2.945 × 10^3^	2.900 × 10^3^
	Worst	3.394 × 10^3^	2.984 × 10^3^	2.947 × 10^3^	3.371 × 10^3^	2.981 × 10^3^	3.060 × 10^3^	2.999 × 10^3^	3.668 × 10^3^	2.998 × 10^3^	3.596 × 10^3^	3.016 × 10^3^	2.900 × 10^3^
	Mean	3.198 × 10^3^	2.954 × 10^3^	2.939 × 10^3^	3.215 × 10^3^	2.957 × 10^3^	2.971 × 10^3^	2.953 × 10^3^	3.574 × 10^3^	2.972 × 10^3^	3.191 × 10^3^	2.970 × 10^3^	2.900 × 10^3^
	WRST	1.201 × 10^−6^/-	△/-	△/-	△/-	△/-	△/-	△/-	△/-	△/-	△/-	△/-	-
	FT	9.550	4.750	2.250	10.300	4.800	5.900	4.400	11.950	6.800	9.700	6.550	1.050
	Rank	10	4	2	11	5	7	3	12	8	9	6	1
Mean Rank		7.167	5.667	3.750	10.833	6.083	7.833	4.083	10.250	4.333	11.000	4.583	2.417
Final Ranking		8	6	2	11	7	9	3	10	4	12	5	1
Mean FT		6.775	6.125	4.654	10.683	5.192	6.775	3.917	10.629	5.004	10.817	4.188	3.242
Final FT		8	7	4	11	6	8	2	10	5	12	3	1
+/=/−		1/1/10	0/2/10	3/2/7	0/0/12	0/2/10	1/0/11	3/4/5	0/0/12	4/1/7	0/0/12	4/3/5	-/-/-

△: 6.796 × 10^−8^ is replaced by △.

**Table 6:** Errors of IA-DTPSO and other MAs on 20-dimensional CEC2022.

F	Index	Algorithms											
		PSO	RUN	NGO	NOA	GKSO	IVYA	EAPSO	G-QPSO	HJSPSO	PSO-Sono	SAO-MPSO	IA-DTPSO
F1	Std	6.898	7.562 × 10^−4^	1.241 × 10^3^	8.173 × 10^3^	8.985 × 10^−4^	3.391 × 10^3^	8.880 × 10^−6^	1.993 × 10^3^	3.761 × 10^2^	1.630 × 10^4^	4.304 × 10^−13^	5.222 × 10^−7^
	RMSE	3.238 × 10^2^	2.990 × 10^2^	4.192 × 10^3^	3.998 × 10^4^	2.995 × 10^2^	1.034 × 10^4^	2.990 × 10^2^	1.883 × 10^4^	1.363 × 10^3^	5.420 × 10^4^	2.990 × 10^2^	2.990 × 10^2^
	*δ*	3.143 × 10^2^	2.990 × 10^2^	2.015 × 10^3^	2.665 × 10^4^	2.990 × 10^2^	5.132 × 10^3^	2.990 × 10^2^	1.452 × 10^4^	6.156 × 10^2^	2.779 × 10^4^	2.990 × 10^2^	2.990 × 10^2^
F2	Std	2.793 × 10^1^	1.797 × 10^1^	1.656 × 10^1^	1.700 × 10^2^	2.180 × 10^1^	1.055 × 10^1^	1.948 × 10^1^	5.161 × 10^1^	1.057 × 10^1^	1.316 × 10^2^	1.585 × 10^1^	3.711
	RMSE	4.427 × 10^2^	4.411 × 10^2^	4.529 × 10^2^	1.057 × 10^3^	4.397 × 10^2^	4.513 × 10^2^	4.383 × 10^2^	1.025 × 10^3^	4.537 × 10^2^	9.290 × 10^2^	4.455 × 10^2^	4.228 × 10^2^
	*δ*	4.144 × 10^2^	3.990 × 10^2^	3.992 × 10^2^	8.046 × 10^2^	3.990 × 10^2^	4.279 × 10^2^	3.990 × 10^2^	9.082 × 10^2^	4.439 × 10^2^	7.166 × 10^2^	4.016 × 10^2^	4.095 × 10^2^
F3	Std	9.017	8.651	7.371 × 10^−2^	1.211 × 10^1^	7.627	6.064	9.377 × 10^−4^	3.797	8.564 × 10^−1^	9.456	7.621 × 10^−1^	4.275
	RMSE	6.328 × 10^2^	6.267 × 10^2^	5.990 × 10^2^	6.576 × 10^2^	6.174 × 10^2^	6.011 × 10^2^	5.990 × 10^2^	6.620 × 10^2^	5.996 × 10^2^	6.501 × 10^2^	5.994 × 10^2^	6.053 × 10^2^
	*δ*	6.144 × 10^2^	6.154 × 10^2^	5.990 × 10^2^	6.284 × 10^2^	6.077 × 10^2^	5.990 × 10^2^	5.990 × 10^2^	6.508 × 10^2^	5.990 × 10^2^	6.281 × 10^2^	5.990 × 10^2^	6.016 × 10^2^
F4	Std	1.502 × 10^1^	1.415 × 10^1^	6.527	1.365 × 10^1^	1.409 × 10^1^	1.287 × 10^1^	1.670 × 10^1^	8.125	7.039	1.627 × 10^1^	1.267 × 10^1^	1.231 × 10^1^
	RMSE	8.633 × 10^2^	8.730 × 10^2^	8.386 × 10^2^	9.736 × 10^2^	8.634 × 10^2^	8.646 × 10^2^	8.406 × 10^2^	9.434 × 10^2^	8.305 × 10^2^	9.729 × 10^2^	8.309 × 10^2^	8.522 × 10^2^
	*δ*	8.389 × 10^2^	8.428 × 10^2^	8.237 × 10^2^	9.485 × 10^2^	8.318 × 10^2^	8.418 × 10^2^	8.189 × 10^2^	9.234 × 10^2^	8.185 × 10^2^	9.404 × 10^2^	8.129 × 10^2^	8.309 × 10^2^
F5	Std	3.758 × 10^2^	2.793 × 10^2^	2.020 × 10^2^	5.955 × 10^2^	3.863 × 10^2^	1.738 × 10^2^	5.727	1.492 × 10^2^	1.234 × 10^1^	6.723 × 10^2^	1.766 × 10^2^	2.139
	RMSE	1.554 × 10^3^	1.752 × 10^3^	1.202 × 10^3^	3.133 × 10^3^	1.358 × 10^3^	2.301 × 10^3^	9.005 × 10^2^	2.782 × 10^3^	9.052 × 10^2^	3.237 × 10^3^	1.030 × 10^3^	9.032 × 10^2^
	*δ*	9.009 × 10^2^	1.340 × 10^3^	9.071 × 10^2^	2.246 × 10^3^	9.081 × 10^2^	1.939 × 10^3^	8.990 × 10^2^	2.578 × 10^3^	8.991 × 10^2^	2.378 × 10^3^	8.992 × 10^2^	8.996 × 10^2^
F6	Std	2.008 × 10^4^	7.669 × 10^2^	6.916 × 10^2^	1.425 × 10^8^	7.584 × 10^3^	1.261 × 10^3^	7.327 × 10^3^	3.893 × 10^7^	8.278 × 10^2^	9.403 × 10^7^	7.738 × 10^3^	2.375 × 10^1^
	RMSE	3.505 × 10^4^	3.624 × 10^3^	3.155 × 10^3^	2.790 × 10^8^	1.245 × 10^4^	3.568 × 10^3^	1.144 × 10^4^	1.259 × 10^8^	2.883 × 10^3^	2.244 × 10^8^	1.199 × 10^4^	1.832 × 10^3^
	*δ*	5.702 × 10^3^	1.922 × 10^3^	2.287 × 10^3^	7.281 × 10^7^	1.863 × 10^3^	1.925 × 10^3^	1.929 × 10^3^	3.348 × 10^7^	1.841 × 10^3^	9.198 × 10^7^	1.946 × 10^3^	1.814 × 10^3^
F7	Std	3.216 × 10^1^	2.292 × 10^1^	1.181 × 10^1^	4.638 × 10^1^	2.474 × 10^1^	2.839 × 10^1^	3.994 × 10^1^	9.078	9.092	5.094 × 10^1^	4.697 × 10^1^	8.845
	RMSE	2.099 × 10^3^	2.108 × 10^3^	2.063 × 10^3^	2.208 × 10^3^	2.070 × 10^3^	2.112 × 10^3^	2.062 × 10^3^	2.181 × 10^3^	2.044 × 10^3^	2.243 × 10^3^	2.068 × 10^3^	2.055 × 10^3^
	*δ*	2.047 × 10^3^	2.044 × 10^3^	2.044 × 10^3^	2.142 × 10^3^	2.026 × 10^3^	2.066 × 10^3^	2.020 × 10^3^	2.162 × 10^3^	2.028 × 10^3^	2.167 × 10^3^	2.020 × 10^3^	2.044 × 10^3^
F8	Std	3.882 × 10^1^	4.257	1.411	4.428 × 10^1^	2.676 × 10^1^	9.088 × 10^1^	5.559 × 10^1^	6.503	2.490	7.743 × 10^1^	8.026	3.748
	RMSE	2.242 × 10^3^	2.226 × 10^3^	2.226 × 10^3^	2.299 × 10^3^	2.231 × 10^3^	2.329 × 10^3^	2.263 × 10^3^	2.252 × 10^3^	2.229 × 10^3^	2.376 × 10^3^	2.226 × 10^3^	2.225 × 10^3^
	*δ*	2.224 × 10^3^	2.222 × 10^3^	2.222 × 10^3^	2.249 × 10^3^	2.220 × 10^3^	2.220 × 10^3^	2.220 × 10^3^	2.237 × 10^3^	2.224 × 10^3^	2.238 × 10^3^	2.220 × 10^3^	2.215 × 10^3^
F9	Std	1.829 × 10^−2^	3.946 × 10^−3^	1.702 × 10^−6^	5.092 × 10^1^	9.247 × 10^−5^	2.422 × 10^−1^	1.368 × 10^−12^	5.391 × 10^1^	1.078 × 10^−3^	6.991 × 10^1^	3.306 × 10^−5^	2.286
	RMSE	2.464 × 10^3^	2.480 × 10^3^	2.480 × 10^3^	2.649 × 10^3^	2.480 × 10^3^	2.480 × 10^3^	2.480 × 10^3^	2.808 × 10^3^	2.480 × 10^3^	2.703 × 10^3^	2.480 × 10^3^	2.476 × 10^3^
	*δ*	2.464 × 10^3^	2.480 × 10^3^	2.480 × 10^3^	2.564 × 10^3^	2.480 × 10^3^	2.480 × 10^3^	2.480 × 10^3^	2.711 × 10^3^	2.480 × 10^3^	2.578 × 10^3^	2.480 × 10^3^	2.471 × 10^3^
F10	Std	8.723 × 10^2^	2.812 × 10^1^	2.785 × 10^1^	1.170 × 10^3^	4.710 × 10^1^	8.874 × 10^2^	4.374 × 10^2^	1.884 × 10^1^	3.044 × 10^1^	2.323 × 10^3^	4.664 × 10^2^	1.253 × 10^−1^
	RMSE	3.736 × 10^3^	2.506 × 10^3^	2.506 × 10^3^	3.231 × 10^3^	2.510 × 10^3^	3.311 × 10^3^	2.917 × 10^3^	2.633 × 10^3^	2.507 × 10^3^	5.120 × 10^3^	3.088 × 10^3^	TO
	*δ*	2.499 × 10^3^	TO	2.499 × 10^3^	2.537 × 10^3^	2.499 × 10^3^	2.499 × 10^3^	TO	TO	TO	2.521 × 10^3^	2.499 × 10^3^	2.499 × 10^3^
F11	Std	7.486 × 10^1^	3.072 × 10^1^	9.754 × 10^1^	8.589 × 10^2^	1.058 × 10^2^	4.702 × 10^1^	5.104 × 10^1^	2.046 × 10^2^	1.187 × 10^2^	6.222 × 10^2^	7.807 × 10^−13^	9.178 × 10^1^
	RMSE	2.950 × 10^3^	2.909 × 10^3^	2.888 × 10^3^	5.673 × 10^3^	2.964 × 10^3^	2.929 × 10^3^	2.944 × 10^3^	6.744 × 10^3^	2.886 × 10^3^	4.995 × 10^3^	2.899 × 10^3^	2.923 × 10^3^
	*δ*	2.650 × 10^3^	2.899 × 10^3^	2.599 × 10^3^	4.175 × 10^3^	2.899 × 10^3^	2.899 × 10^3^	2.899 × 10^3^	6.224 × 10^3^	2.599 × 10^3^	3.675 × 10^3^	2.899 × 10^3^	2.599 × 10^3^
F12	Std	1.481 × 10^2^	1.042 × 10^1^	3.139	7.512 × 10^1^	1.007 × 10^1^	2.848 × 10^1^	1.459 × 10^1^	5.104 × 10^1^	1.208 × 10^1^	1.195 × 10^2^	1.768 × 10^1^	1.037 × 10^−4^
	RMSE	3.200 × 10^3^	2.953 × 10^3^	2.938 × 10^3^	3.215 × 10^3^	2.956 × 10^3^	2.970 × 10^3^	2.952 × 10^3^	3.573 × 10^3^	2.971 × 10^3^	3.192 × 10^3^	2.969 × 10^3^	2.899 × 10^3^
	*δ*	2.895 × 10^3^	2.940 × 10^3^	2.934 × 10^3^	3.112 × 10^3^	2.943 × 10^3^	2.946 × 10^3^	2.933 × 10^3^	3.468 × 10^3^	2.953 × 10^3^	3.058 × 10^3^	2.944 × 10^3^	2.899 × 10^3^

**Table 7:** TUWRs in China from 2004 to 2023 (Unit: Hundred million cubic meters).

Years	2004	2005	2006	2007	2008	2009	2010	2011	2012	2013
TUWRs	24,129.6	28,053.1	25,330.1	25,255.2	27,434.3	24,180.2	30,906.4	23,256.7	29,528.8	27,957.9
Years	2014	2015	2016	2017	2018	2019	2020	2021	2022	2023
TUWRs	27,266.9	27,962.6	32,466.4	28,761.2	27,462.5	29,041.0	31,605.2	29,638.2	27,088.1	24,780.0

**Table 8:** Statistical results of IA-DTPSO and other MAs for addressing TUWRs in China.

Years	Real Value	IA-DTPSO			PSO			GKSO			IVYA		
		SimD	ResE	APE (%)	SimD	ResE	APE (%)	SimD	ResE	APE (%)	SimD	ResE	APE (%)
2005	28,053.1	27,916.57	−136.53	0.49	24,398.68	−3654.42	13.03	26,529.37	−1523.73	5.43	25,472.86	−2580.23	9.19
2006	25,330.1	25,594.21	264.11	1.04	25,454.02	123.92	0.49	26,220.56	890.46	3.52	25,966.25	636.15	2.51
2007	25,255.2	25,617.06	361.86	1.43	26,417.97	1162.77	4.60	26,288.93	1033.73	4.09	26,595.10	1339.90	5.30
2008	27,434.3	26,010.91	−1423.39	5.19	27,180.11	−254.19	0.93	26,494.88	−939.41	3.42	27,052.92	−381.37	1.39
2009	24,180.2	26,496.10	2315.90	9.58	27,742.49	3562.29	14.73	26,770.27	2590.07	10.71	27,386.22	3206.02	13.25
2010	30,906.4	26,978.82	−3927.58	12.71	28,130.40	−2776.00	8.98	27,071.13	−3835.27	12.41	27,628.87	−3277.52	10.60
2011	23,256.7	27,423.85	4167.15	17.92	28,372.31	5115.61	22.00	27,373.44	4116.74	17.70	27,805.52	4548.82	19.55
2012	29,528.8	27,818.17	−1710.63	5.79	28,494.53	−1034.27	3.50	27,665.48	−1863.32	6.31	27,934.13	−1594.66	5.40
2013	27,957.9	28,158.21	200.31	0.72	28,519.84	561.94	2.01	27,942.35	−15.55	0.06	28,027.75	69.85	0.24
2014	27,266.9	28,444.74	1177.84	4.32	28,467.42	1200.52	4.40	28,202.71	935.81	3.43	28,095.92	829.02	3.04
2015	27,962.6	28,680.48	717.88	2.57	28,353.20	390.60	1.40	28,446.89	484.29	1.73	28,145.54	182.94	0.65
2016	32,466.4	28,869.04	−3597.36	11.08	28,190.29	−4276.11	13.17	28,675.96	−3790.44	11.67	28,181.67	−4284.72	13.19
2017	28,761.2	29,014.29	253.09	0.88	27,989.42	−771.78	2.68	28,891.27	130.07	0.45	28,207.97	−553.22	1.92
2018	27,462.5	29,120.10	1657.60	6.04	27,759.33	296.83	1.08	29,094.15	1631.65	5.94	28,227.12	764.62	2.78
MAPE*_simulation_* (%)		5.6366			6.6432			6.2061			6.3627		
2019	29,041	29,106.99	65.99	0.23	28,640.37	−400.63	1.3795	29,285.85	244.85	0.84	28,241.06	−799.93	2.75
2020	31,605.2	29,112.08	−2493.12	7.89	28,817.00	−2788.20	8.8220	29,467.49	−2137.70	6.76	28,251.21	−3353.98	10.61
2021	29,638.2	29,086.75	−551.45	1.86	29,009.75	−628.45	2.1204	29,640.08	1.88	0.01	28,258.59	−1379.60	4.65
2022	27,088.1	29,034.33	1946.23	7.18	29,216.74	2128.64	7.8582	29,804.48	2716.37	10.02	28,263.97	1175.87	4.34
2023	24,780	28,957.86	4177.86	16.86	29,436.13	4656.13	18.7899	29,961.43	5181.43	20.90	28,267.89	3487.89	14.07
MAPE*_prediction_* (%)		6.8041			7.2254			7.7102			7.2876		
MAPE (%)		5.9439			6.7964			6.6019			6.6061		
**Years**	**Real Value**	**EAPSO**			**HJSPSO**			**PSO-sono**			**SAO-MPSO**		
		**SimD**	**ResE**	**APE (%)**	**SimD**	**ResE**	**APE (%)**	**SimD**	**ResE**	**APE (%)**	**SimD**	**ResE**	**APE (%)**
2005	28,053.1	28,036.28	−16.81	0.06	27,865.98	−187.11	0.66	27,772.07	−281.02	1.00	27,744.11	−308.98	1.10
2006	25,330.1	24,823.89	−506.20	1.99	26,510.91	1180.81	4.66	26,521.71	1191.61	4.70	25,728.56	398.46	1.57
2007	25,255.2	25,798.03	542.83	2.14	25,896.87	641.67	2.54	25,936.51	681.31	2.69	26,184.17	928.97	3.67
2008	27,434.3	26,288.65	−1145.64	4.17	25,813.99	−1620.30	5.90	25,854.32	−1579.97	5.75	26,590.81	−843.48	3.07
2009	24,180.2	26,745.13	2564.93	10.60	26,043.52	1863.32	7.70	26,074.90	1894.70	7.83	26,952.85	2772.65	11.46
2010	30,906.4	27,137.43	−3768.96	12.19	26,433.89	−4472.50	14.47	26,454.43	−4451.96	14.40	27,270.65	−3635.74	11.76
2011	23,256.7	27,484.06	4227.36	18.17	26,897.13	3640.43	15.65	26,907.61	3650.91	15.69	27,546.62	4289.92	18.44
2012	29,528.8	27,794.22	−1734.57	5.87	27,385.82	−2142.97	7.25	27,387.58	−2141.21	7.25	27,784.27	−1744.52	5.90
2013	27,957.9	28,075.01	117.11	0.41	27,875.64	−82.25	0.29	27,869.90	−87.99	0.31	27,987.46	29.56	0.10
2014	27,266.9	28,331.62	1064.72	3.90	28,354.76	1087.86	3.98	28,342.48	1075.58	3.94	28,159.99	893.09	3.27
2015	27,962.6	28,568.01	605.40	2.16	28,817.97	855.37	3.05	28,799.80	837.20	2.99	28,305.45	342.85	1.22
2016	32,466.4	28,787.22	−3679.17	11.33	29,263.39	−3203.00	9.86	29,239.83	−3226.56	9.93	28,427.14	−4039.25	12.44
2017	28,761.2	28,991.69	230.49	0.80	29,690.86	929.66	3.23	29,662.25	901.05	3.13	28,528.04	−233.15	0.81
2018	27,462.5	29,183.33	1720.83	6.26	30,100.99	2638.49	9.60	30,067.59	2605.09	9.48	28,610.82	1148.32	4.18
MAPE*_simulation_* (%)		5.7233			6.3508			6.3688			5.6466		
2019	29,041	29,363.72	322.72	1.11	30,494.74	1453.74	5.00	30,456.75	1415.75	4.87	28,677.83	−363.16	1.25
2020	31,605.2	29,534.15	−2071.04	6.55	30,873.19	−732.01	2.31	30,830.78	−774.41	2.45	28,731.17	−2874.02	9.09
2021	29,638.2	29,695.73	57.53	0.19	31,237.40	1599.20	5.39	31,190.71	1552.51	5.23	28,772.67	−865.52	2.92
2022	27,088.1	29,849.35	2761.25	10.19	31,588.41	4500.31	16.61	31,537.54	4449.44	16.42	28,803.96	1715.86	6.33
2023	24,780	29,995.80	5215.80	21.04	31,927.15	7147.15	28.84	31,872.23	7092.23	28.62	28,826.43	4046.43	16.32
MAPE*_prediction_* (%)		7.8201			11.6347			11.522			7.1856		
MAPE (%)		6.2751			7.7413			7.7249			6.0516		

**Table 9:** Parameter statistics of IA-DTPSO and other algorithms for solving the TUWRs in China.

Parameters	IA-DTPSO	PSO	GKSO	IVYA	EAPSO	HJSPSO	PSO-Sono	SAO-MPSO
*Csz*	24,123.6	24,160.2	24,385.5	24,346.2	24,488.9	24,146.9	24,500	24,245.4
*ξ*	0.080129	0.560555	0.377924	0.439050	0.198431	0.029187	0.345376	0.067554
*r*	1.453199	0.360578	0.927702	0.899874	0.905127	0.937307	1	0.925674
*a*	0.058716	0.17176	0.64349	0.27198	1.4176	0.64964	0.64406	0.12418
*b*	6780.2789	−39.7023	13,448.3342	7691.1691	30,532.6828	9443.9293	9426.1429	4152.6776
*c*	23,519.5322	8199.7156	20,248.424	16,383.655	6180.5493	24,060.5466	24,153.8696	21,550.6303

**Table 10:** Prediction results of TUWRs in China in the next five years.

Years	2024	2025	2026	2027	2028
TUWRs	26,376.97	26,028.78	24,960.55	28,731.54	33,688.46

**Table 11:** Statistical results of solving TUWRs in China using different models.

Years	Real Value	ID_T			GM(1,1)			DGM(1,1)			NGBM(1,1)		
		SimD	ResE	APE (%)	SimD	ResE	APE (%)	SimD	ResE	APE (%)	SimD	ResE	APE (%)
2005	28,053.1	27,916.57	−136.53	0.49	25,987.99	−2065.11	7.36	26,023.11	−2029.99	7.24	25,987.99	−2065.11	7.36
2006	25,330.1	25,594.21	264.11	1.04	26,221.03	890.93	3.52	26,251.27	921.17	3.64	26,221.03	890.93	3.52
2007	25,255.2	25,617.06	361.86	1.43	26,456.15	1200.95	4.76	26,481.43	1226.23	4.86	26,456.15	1200.95	4.76
2008	27,434.3	26,010.91	−1423.39	5.19	26,693.38	−740.92	2.70	26,713.60	−720.70	2.63	26,693.38	−740.92	2.70
2009	24,180.2	26,496.10	2315.90	9.58	26,932.74	2752.54	11.38	26,947.82	2767.62	11.45	26,932.74	2752.54	11.38
2010	30,906.4	26,978.82	−3927.58	12.71	27,174.25	−3732.15	12.08	27,184.08	−3722.32	12.04	27,174.25	−3732.15	12.08
2011	23,256.7	27,423.85	4167.15	17.92	27,417.92	4161.22	17.89	27,422.42	4165.72	17.91	27,417.92	4161.22	17.89
2012	29,528.8	27,818.17	−1710.63	5.79	27,663.78	−1865.02	6.32	27,662.85	−1865.95	6.32	27,663.78	−1865.02	6.32
2013	27,957.9	28,158.21	200.31	0.72	27,911.84	−46.06	0.16	27,905.39	−52.51	0.19	27,911.84	−46.06	0.16
2014	27,266.9	28,444.74	1177.84	4.32	28,162.12	895.22	3.28	28,150.05	883.15	3.24	28,162.12	895.22	3.28
2015	27,962.6	28,680.48	717.88	2.57	28,414.65	452.05	1.62	28,396.85	434.25	1.55	28,414.65	452.05	1.62
2016	32,466.4	28,869.04	−3597.36	11.08	28,669.45	−3796.95	11.70	28,645.83	−3820.57	11.77	28,669.45	−3796.95	11.70
2017	28,761.2	29,014.29	253.09	0.88	28,926.52	165.32	0.57	28,896.98	135.78	0.47	28,926.52	165.32	0.57
2018	27,462.5	29,120.10	1657.60	6.04	29,185.91	1723.41	6.28	29,150.34	1687.84	6.15	29,185.91	1723.41	6.28
MAPE*_simulation_* (%)		5.6366			6.4009			6.3887			6.4005		
2019	29,041	29,106.99	65.99	0.23	29,447.62	406.62	1.40	29,405.91	364.91	1.26	29,447.62	406.62	1.40
2020	31,605.2	29,112.08	−2493.12	7.89	29,711.67	−1893.53	5.99	29,663.73	−1941.47	6.14	29,711.67	−1893.53	5.99
2021	29,638.2	29,086.75	−551.45	1.86	29,978.10	339.90	1.15	29,923.81	285.61	0.96	29,978.10	339.90	1.15
2022	27,088.1	29,034.33	1946.23	7.18	30,246.91	3158.81	11.66	30,186.17	3098.07	11.44	30,246.91	3158.81	11.66
2023	24,780	28,957.86	4177.86	16.86	30,518.14	5738.14	23.16	30,450.83	5670.83	22.88	30,518.14	5738.14	23.16
MAPE*_prediction_* (%)		6.8041			8.6711			8.5370			8.5839		
MAPE (%)		5.9439			6.9983			6.9540			6.9751		

**Table 12:** Prediction results of TUWRs in China for the next five years under four different models.

Years	2024	2025	2026	2027	2028
ID_T	26,376.97	26,028.78	24,960.55	28,731.54	33,688.46
GM(1,1)	30,791.79	31,067.90	31,346.48	31,627.57	31,911.17
DGM(1,1)	30,717.80	30,987.12	31,258.80	31,532.87	31,809.33
NGBM(1,1)	29,691.42	29,811.48	29,928.85	30,043.79	30,156.50

## Data Availability

All data generated or analyzed during the study are included in this published article.
